# A spatiotemporal atlas of hydropower in Africa for energy modelling purposes

**DOI:** 10.12688/openreseurope.13392.3

**Published:** 2022-03-29

**Authors:** Sebastian Sterl, Albertine Devillers, Celray James Chawanda, Ann van Griensven, Wim Thiery, Daniel Russo

**Affiliations:** 1 Department of Hydrology and Hydraulic Engineering, Vrije Universiteit Brussel, Brussels, 1050, Belgium; 2 Department of Earth and Environmental Sciences, KU Leuven, Leuven, 3001, Belgium; 3 Center for Development Research (ZEF), University of Bonn, Bonn, 53113, Germany; 4 International Renewable Energy Agency (IRENA), Bonn, 53113, Germany; 5 Mines ParisTech, Paris, 75272, France; 6 IHE-Delft Institute for Water Education, Westvest 7, Delft, 2611AX, The Netherlands

**Keywords:** Hydropower, energy modelling, Africa, resource profiles, renewables, decarbonization

## Abstract

The modelling of electricity systems with substantial shares of renewable resources, such as solar power, wind power and hydropower, requires datasets on renewable resource profiles with high spatiotemporal resolution to be made available to the energy modelling community. Whereas such resources exist for solar power and wind power profiles on diurnal and seasonal scales across all continents, this is not yet the case for hydropower. Here, we present a newly developed open-access African hydropower atlas, containing seasonal hydropower generation profiles for nearly all existing and several hundred future hydropower plants on the African continent. The atlas builds on continental-scale hydrological modelling in combination with detailed technical databases of hydropower plant characteristics and can facilitate modelling of power systems across Africa.

## Plain language summary

Hydropower plants rely on river flow to generate electricity. Since river flows change between different seasons, electricity from hydropower plants will also change from season to season. In this paper, we present a new database that contains calculated profiles of electricity generation from season to season for hundreds of hydropower plants in Africa, both existing and future ones. This database will be helpful to scientists doing research on electricity generation in different African countries.

## 1. Introduction

To achieve the long-term objectives of the Paris Agreement, it is well-established that electricity supply worldwide will have to decarbonise by mid-century ^1^. In this context, it is imperative that the shares of low-carbon resources in power systems increase. Low-carbon resources include solar photovoltaics (PV), concentrated solar power (CSP), wind power, hydropower, geothermal power, ocean power, bioenergy and nuclear power. Among these, the strongest growth rates over the past decade, and the highest drops in price, have been recorded by solar PV and wind power ^2^, which are thus seen more and more as potential backbones of future power systems ^3^.

Given the dependence of solar PV and wind power generation on meteorological variables, these are classified as “variable renewables”, or VRE ^4^. Because of this variability in generation from short (sub-hourly) to long (seasonal and interannual) timescales, increasing the share of VRE in electricity systems will require increased flexibility and storage to solve issues related to mismatches between VRE supply and electricity demand, which must be considered in modelling exercises ^5^.

Although solar and wind power have recorded the highest rates of growth among renewable resources in recent years, the most-used renewable electricity resource worldwide is currently still hydropower ^2^. This comprises run-of-river hydropower without storage, which is essentially another form of VRE ^6^; reservoir hydropower, which can be dispatched flexibly to aid VRE grid integration ^4, 7– 11^; and pumped-storage hydropower, which can be used as a “battery” to avoid curtailment of surplus VRE ^12^.

To inform long-term planning and modelling of renewable power capacity expansion, it is crucial that reliable resource profiles of VRE and hydropower are available to the modelling community ^13^. The inclusion of such resource profiles at high spatiotemporal resolution, from hourly to seasonal and interannual timescales and across geospatial zones of different resource strengths, is crucial to accurately represent modern renewable technologies in energy system models. For this reason, dedicated spatiotemporal databases on solar and wind resource strength and availability have been developed, such as the Global Solar Atlas ^14^ and the Global Wind Atlas ^15^ or the reanalysis-based web interface “ *renewables.ninja*” ^16^. Such resources typically allow the user to select locations on the world map and extract representative resource profiles for VRE from hourly to seasonal and interannual timescales, which can then be used in energy modelling exercises.

The picture is different for hydropower. Comprehensive and integrated databases of hydropower resources are currently unavailable to the modelling community at the required level of detail ^17^. This is a consequence of the challenge of accurately modelling river flows across a wide range of river basins with different hydrometeorological conditions within a single model framework ^18^, as well as the wide disparity in individual hydropower plants’ technical characteristics ^19^. A consequence of this comparative disparity vis-à-vis solar and wind power, and the resulting lack of comprehensive hydropower databases, is that hydropower plants – which are more and more considered to be an important lever to support VRE uptake thanks to their flexibility of dispatch (for reservoir plants) and potential seasonal synergy with VRE (for run-of-river plants) – are often represented coarsely and without the warranted spatiotemporal detail in energy models ^9^. For instance, many studies lump hydropower plants in a region together as one single technology without detail on individual plants (e.g. 3, 20), do not consider interannual variability of river flows (e.g. 21), or do not contain information on seasonally constrained availabilities of hydropower (e.g. 22).

This data gap is especially problematic for regions where (i) hydropower forms an important backbone of many power systems, (ii) substantial expansions of hydropower generation are still planned, and (iii) precipitation patterns are highly variable on seasonal timescales. All of these apply to the African continent ^23– 25^, for which science-based services for the renewable energy sector are in short supply ^26^. To close the data gap and improve the resources available for energy modelling on Africa, we present here a new spatiotemporal data atlas for nearly all existing and several hundred future hydropower plants across the African continent, containing (i) geospatial references, (ii) technical characteristics, and (iii) seasonal power plant availability profiles, including uncertainty ranges reflecting interannual hydrological variability. The seasonal availability profiles in the atlas include the effect of reservoir sizes on operational possibilities to shift seasonal availabilities of hydropower dispatch, and of current and future configurations of hydropower plants in a cascade. This African hydropower atlas is hereafter abbreviated by “AHA”.

## 2. Materials and methods

The AHA, which is herewith made freely available to the research community, is designed to be a comprehensive resource containing technical, spatial, and temporal data on existing and future hydropower plants across Africa. It covers all continental African countries which together constitute the major African Power Pools (respectively the North, West, Central, Eastern, and Southern African Power Pool), as well as the island nation of Madagascar.

**Figure 1.**
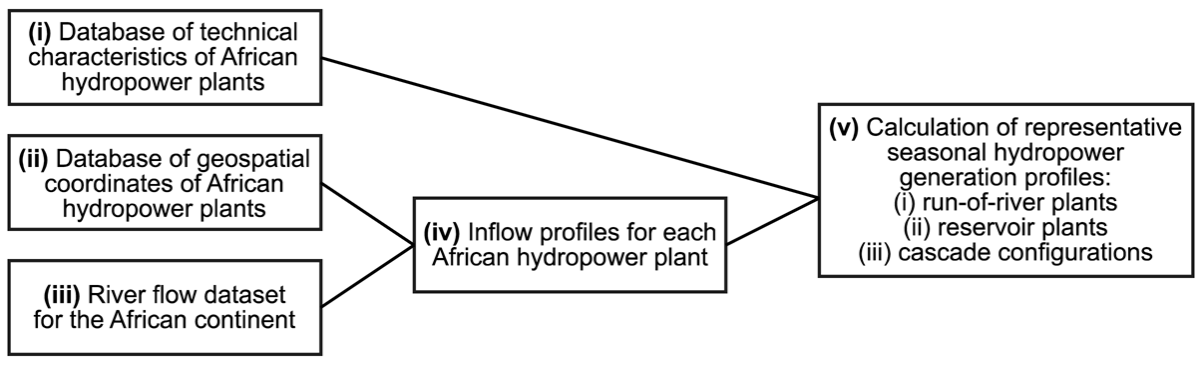
Schematic overview of the various inputs, intermediate results, and outputs of the calculations performed to create the African Hydropower Atlas.

The AHA is collated into a single spreadsheet-based file which contains both inputs and results of the calculations carried out to establish the atlas. An overview of the calculation flow performed to obtain the full dataset is provided in Figure 1. Each of the elements of this workflow are described in a separate subsection hereafter.

### 2.1 Database of technical characteristics of African hydropower plants

The technical information for each hydropower plant includes the rated capacity (in MW), the reservoir size (in million m ^3^ wherever applicable), the multiannual mean discharge of the river section upon which the plant is located (in m ^3^/s), the design discharge wherever known (in m ^3^/s), the earliest expected year of entry into service, and the multiannual average capacity factor of the plant wherever known from previous research (in %). Here, the capacity factor of any power plant is taken to refer to the electricity that it generated over a certain time period (e.g. hour, day, season, year...) divided by the power that would have been generated if the plant had run at full capacity over that entire period. In cases where the latter value was unknown, it was assumed to be 50% based on typical values observed for hydropower plants around the world ^2^.

This data was collated from a wide array of available information. Globally, the data sources can be divided into three categories: (i) existing hydro databases, such as the Global Reservoir and Dam (GRanD) database ^27^, the FAO’s Dams in Africa dataset ^28^, and the West African Renewable Power Database (WARPD) ^9^; (ii) bespoke information, pertaining to individual hydropower projects, from technical project overview sheets, environmental impact assessments, white papers, scientific papers, and other technical modelling studies; and (iii) online news articles on hydropower projects. The consultation and selection of data sources happened strictly according to the hierarchy (i)-(ii)-(iii), with sources from category (i) forming the default, being supplemented by categories (ii) and (iii) wherever necessary. All used data sources are referenced in the AHA. The processing of this data to calculate temporal hydropower availability profiles is explained further below, in section 2.5.

The database includes both existing (active) hydropower plants, as well as future plants. The term “future” is relatively broad and may encompass, for example, projects under construction or in the pipeline, projects in need of financing, or projects in the pre-feasibility phase. In many cases, distinguishing between these categories is not straightforward. Based on the above-mentioned data sources, the AHA distinguishes between three categories of future projects in descending order of concreteness: committed, planned, and candidate. For any future plant where no specific information was identified regarding its status (as of the writing of this paper), the categorization was set to “candidate” by default. In those cases, the “first year” parameter was left empty. Projects in this category may either be currently unlikely to obtain financing, have been shelved, or have never gone beyond pre-feasibility studies.

We note that we constrained the entries to the current version of the atlas by the criterion that the data should be available in publicly consultable sources. Thus, the atlas could be improved if presently undisclosed information available in, for example, internal documents of planning agencies were to be made publicly available. We therefore eagerly invite all relevant stakeholders to review and submit corrections and/or missing data to the author team, since the goal is for the database to be regularly updated. This particularly concerns the list of future projects, which can likely be expanded much beyond its current state and of which we do not claim full comprehensiveness.

**Figure 2.**
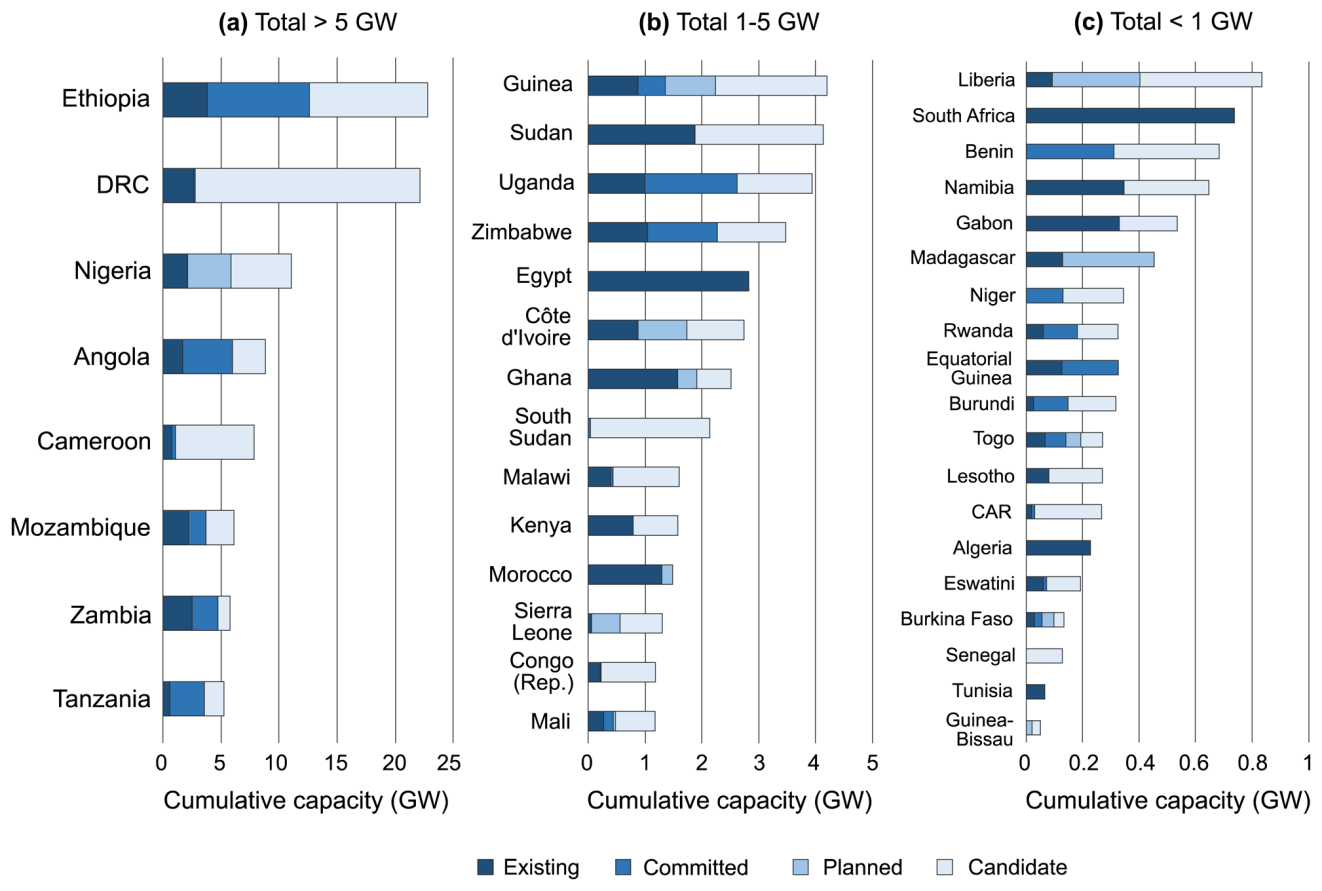
Overview of total capacity of existing, committed, planned, and candidate hydropower plants across Africa as collected in the AHA, for countries where this capacity totals ( **a**) > 5 GW, ( **b**) 1-5 GW, and ( **c**) < 1 GW. DRC = Democratic Republic of the Congo; Congo (Rep.) = Republic of the Congo; CAR = Central African Republic.

Currently, the AHA contains a total of 633 entries on hydropower plants, of which 266 are existing, 60 committed, 44 planned and 263 candidates. Their total capacity amounts to 132 GW, of which 24% is existing (approximately 32 GW, lining up well with other statistics on existing plants ^29^), 19% committed, 6% planned, and the remaining 51% candidate. The division of the total capacity by category and by country is shown in Figure 2.

We note that hydropower plants have been allocated to the country of their coordinates, notwithstanding that, in some cases, a part of the produced electricity would be allocated for exports (e.g. hydropower plants in some river basins are shared among all riparian countries). In the cases of hydropower plants located on rivers forming country borders (11 cases in total in the AHA), their capacity was allocated equally among the countries in question, thus forming separate entries in the database.

### 2.2 Database of geospatial coordinates of African hydropower plants

The geo-referencing of hydropower plants was done according to a hierarchy of data choices, depending on the status of each plant. Firstly, all existing plants were georeferenced using satellite imagery; the coordinates given in the AHA correspond to the location of the dam and/or powerhouse as identifiable via Google Maps. Secondly, all hydropower plants that are not yet servicing the grid but are clearly identifiable as being under construction on satellite imagery, were similarly georeferenced. Thirdly, the locations of all other committed, planned and candidate hydropower plants were identified as best possible from specific project information available in any of the consulted sources.

This last category of data could take on a variety of specificity: in some cases, georeferenced coordinates of the intended location of the planned plant were provided in the consulted document(s) as referenced in the AHA; in others, the information remained less precise (e.g. “ *the plant will be constructed about 50 km downstream of location A, about 100 km west of city B*”). In the latter case, satellite imagery was consulted to roughly identify the river section corresponding to the description, and a “best guess” location (e.g. where whitewater reveals the presence of rapids, showing a relatively steep head drop) was selected on the river section. We note that, as long as the river section is identifiable at the spatial resolution of the river flow data that is used (see section 2.3), this approximation is unproblematic for the analysis.

**Figure 3.**
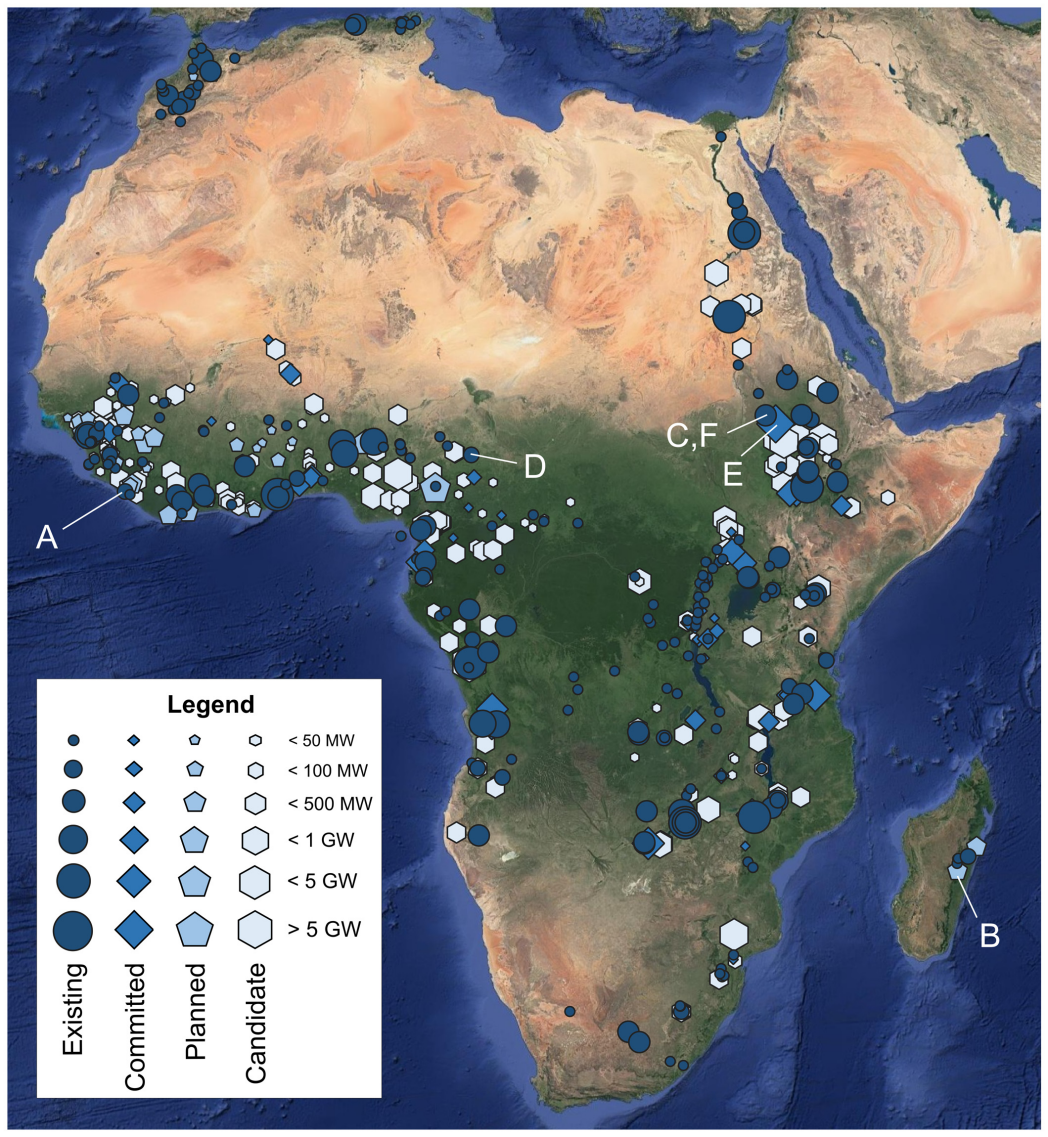
An overview of the georeferenced African hydropower plants by category (existing, committed, planned, candidate). Sizes of icons reflect installed capacity as per the legend. The characters ( **A**)–( **F**) refer to the plants whose temporal power generation profiles are shown in Figure 4. Background: Esri’s World Imagery ^32^ (see Acknowledgements).

A spatial overview of the hydropower plants collected in the AHA is shown in Figure 3.

### 2.3 River flow dataset for the African continent

To estimate hydropower generation profiles for each of the identified locations under the given technical plant characteristics, estimations of river flow at monthly resolution on the African continent were obtained from dedicated simulations with SWAT+ (Soil and Water Assessment Tool ^30^). A previous version of this dataset has been used for hydropower potential assessment in West Africa before (refs. 9, 31); the updated version used for this paper is available through the repository in ref. 33. Detailed descriptions of the characteristics of the simulations are provided in refs. 9, 34, 35; performance metrics of the simulations in comparison to observed data from the Global Runoff Data Centre (GRDC) are described in ref. 35. The most important points from these publications are repeated below.

In SWAT+, watersheds are delineated into sub-basins from which hydrologic response units (HRUs, which are distinct areas of a sub-basin with a unique combination of land use, soil type and slope class) are defined. For the SWAT+ model used for the AHA, sub-basins were delineated using 3,500 km ^2^ as threshold, yielding 5,700 sub-basins and 461,829 HRUs across the African continent. Input data was obtained from the following sources:

➢ Digital elevation: A 90 × 90 m Digital Elevation Model (DEM) acquired from the Shuttle Radar Topography Mission ^36^;
➢ Land use: Data from the Land Use Harmonization (LUH2) dataset ^37^ at 0.25° × 0.25° resolution;
➢ Irrigated areas were obtained from Food and Agriculture Organisation (FAO) data at 0.083° × 0.083° resolution ^38^. Irrigation modelling was implemented as explained in ref. 35
➢ Soil: Data from the Africa Soil Information Service (AfSIS) dataset ^39^ resampled at 0.25° × 0.25°;
➢ Meteorological forcing: Data from the EWEMBI dataset ^40^ at 0.5° × 0.5°.

Further, the following methodologies were employed to estimate evapotranspiration and surface runoff and perform flow routing:

➢ Evapotranspiration: Using the Penman–Monteith method ^41^;
➢ Surface runoff: Using the Soil Conservation Service curve number method ^42^;
➢ Flow routing: Using the variable storage routing method ^43^.

Temporally, the simulations were carried out at daily resolution across the 37-year period 1980–2016. For the reposited dataset, results were averaged to monthly timescales to reduce file size. The first eight years of the simulation were considered as spin-up time and left out of the analysis. Spatially, each river section of the modelled river network is designated by a unique identifier (ID) as provided in the reposited dataset, to which hydropower plant coordinates could be mapped (see next section).

### 2.4 Inflow profiles for each African hydropower plant

The geospatial information described in section 2.2 and the river flow information described in section 2.3 were combined as follows to obtain the river inflow feeding each hydropower plant.

First, the geospatial hydropower plant information (coordinates) was mapped to the river network of the SWAT+ simulations (river sections), such that monthly river flow across the 37-year simulation period could be extracted separately for each hydropower plant. This “snapping” was straightforward in 74% of cases, with hydropower plant coordinates being precisely covered by the SWAT+ river network. In the other 26% of cases, the river stretch most representative for the hydropower plant coordinates was selected according to the following hierarchy. First, if the hydropower plant coordinates were so close to the river source that the modelled SWAT+ network did not extend sufficiently far upstream, the most upstream river section in the modelled network (downstream of the plant coordinates) was selected. Second, if the hydropower plant was located on an affluent not covered by the SWAT+ network at all, the geographically nearest river section in the same river basin (draining into the same main river) was selected. Third, in the extremely rare cases where the entire river basin of the hydropower plant was not covered by the SWAT+ network, but a nearby river basin with the same prevalent precipitation seasonality was covered, the geographically nearest river section of that basin was selected. Note that in all these cases, the objective of this snapping was to infer a reasonable estimate of river flow seasonality and interannual variability for each hydropower plant. The AHA includes the selected SWAT+ river section ID for each identified set of hydropower plant coordinates.

Second, a typical range of seasonal profiles of different “wetness”, spanning the range from very dry to very wet years, was selected as follows. First, the flow profile for a “normal year” was defined as the monthly median of the dataset. Subsequently, the flow profile for “very dry” and “very wet” years was taken to be the “normal year” profile multiplied by a corrective factor, calculated as the ratio of the 5 ^th^ (very dry) and 95 ^th^ (very wet) percentile value of average annual flow to the multiannual average flow. To account for the fact that some few hydropower plants with very large reservoirs are capable of buffering water on interannual timescales and thus mitigate interannual variability, an exception in the calculation was made for those plants with a typical filling time ^9^ of more than one full year. For these plants, instead of the 5 ^th^ and 95 ^th^ percentiles, the 10 ^th^ and 90 ^th^ percentiles were taken to account for this mitigation of dry and wet extremes on interannual timescales.

Third, the seasonal river flow profile thus obtained (for very dry, normal, and very wet years), each characterised as a time series of twelve values representing the months of the year, was normalised to dimensionless values by dividing each time series by the simulated multiyear average flow as simulated (a single number). In this way, the (normalized) seasonality was obtained for each plant in the AHA for which a match of geospatial coordinates with SWAT+ simulated river stretches could be performed.

Fourth, wherever possible, the three resulting dimensionless time series of normalised river inflow to each hydropower plant were multiplied again with a bias-correction factor (simple scaling ^44^), equal to the multiannual mean river discharge value collected from existing databases and literature (again, a single number; see section 2.1). Bias-correction was only performed for cases where either (i) mean discharge values were available for the hydropower site in question (e.g. from environmental impact assessments where measurements directly at the hydropower plant construction site were invoked), or (ii) mean discharge values were available from gauging stations located at or very close to the hydropower plant. (Note that we did not work with a specific reference period for the bias-correction, which was meant rather as an estimation to be compared to design discharge than as a highly accurate measure of absolute flow rates, since this would have been near-impossible to apply across the board given the disparity in observational data sets.) In this way, bias-correction could be applied to 60% of cases (380 out of 633 plants). We note that this step is not crucial for the data product and serves mostly for providing a dimension to the river flow seasonality (expressing it in m ^3^/s instead of dimensionless, which allows a comparison to e.g. design discharges of hydropower plants), as the monthly availability curves of hydropower plants could still be estimated without performing the bias-correction step, i.e. for the remaining 40% of cases (see section 2.5.1).

### 2.5 Calculation of representative seasonal hydropower availability profiles for energy modelling

The final step in the calculations was to convert the typical river inflow datasets (whether bias-corrected or not) for each reservoir to typical power output profiles. A distinction was made between (i) run-of-river hydropower plants, (ii) reservoir hydropower plants, and (iii) hydropower plants in a cascade. For each of these, typical profiles of outflow (e.g. of turbined water) were calculated from inflow profiles as described below, before these were further converted to typical seasonal capacity factors.

***2.5.1 Run-of-river hydropower plants.*** For run-of-river hydropower plants, the turbined outflow profiles were taken equal to the inflow profiles. Power generation was assumed to be a linear function of the turbined outflow profile, with the exception that maximum power output was assumed to be reached when outflow was equal to or higher than the design discharge (reflecting the fact that run-of-river hydropower plants are typically designed to produce at full capacity during several months of the year, not only during the single wettest month). Typical seasonal capacity factors were thus calculated according to:

$${\langle CF_{hydro}\rangle }_{m}^{n,d,w}\;=\;\min\left(\frac{{\langle Q(t)\rangle }_{m}^{n,d,w}}{Q_{design}},1\right),\;(1)$$

where ${\langle CF_{hydro}\rangle }_{m}^{n,d,w}$ is the average capacity factor of the hydropower plant in month *m* during a normal ( *n*), very dry ( *d*) or very wet ( *w*) year; ${\langle Q(t)\rangle }_{m}^{n,d,w}$ is the average turbined outflow in that month; and *Q _design_* is the design discharge.

In cases where the design discharge was not known, it was estimated by dividing the multiannual mean river discharge value (used for bias-correction of SWAT+ data) by the multiannual average capacity factor recorded in the AHA (assumed to be 50% unless known otherwise, as mentioned in section 2.1). Thus, for instance, the design discharge of a hydropower plant with an average capacity factor of 50% was assumed to be twice the average discharge. For such cases, the capacity factor was thus calculated according to:

$${\langle CF_{hydro}\rangle }_{m}^{n,d,w}=\;\min\left(\frac{\langle Q{\left(t\right)}_{m}^{n,d,w}\rangle }{Q_{mean}}\times CF_{hydro}^{mean},1\right),\;(2)$$

where $CF_{hydro}^{mean}$ is the assumed multiannual average capacity factor, and *Q _mean_* the multiannual average river discharge.

In those cases where neither the design discharge *Q _design_* nor the multiannual mean river discharge *Q _mean_* were available (the latter meaning that no bias-correction could be performed), it was assumed that the design discharge corresponded to 50% of the maximum monthly flow in a “normal” year. The (non-bias corrected) annual cycles were then divided by that (non-bias corrected) value, thus obtaining an estimate of typical monthly average capacity factors:

$${\langle CF_{hydro}\rangle }_{m}^{n,d,w}\;=\;\min\left(\frac{{\langle q(t)\rangle }_{m}^{n,d,w}}{0.5\;\times \;\underset{m}{\max}[{\langle q(t)\rangle }_{m}^{n}]},1\right),\;(3)$$

where *q*( *t*) represents the flow time series before bias-correction.

**Figure 4.**
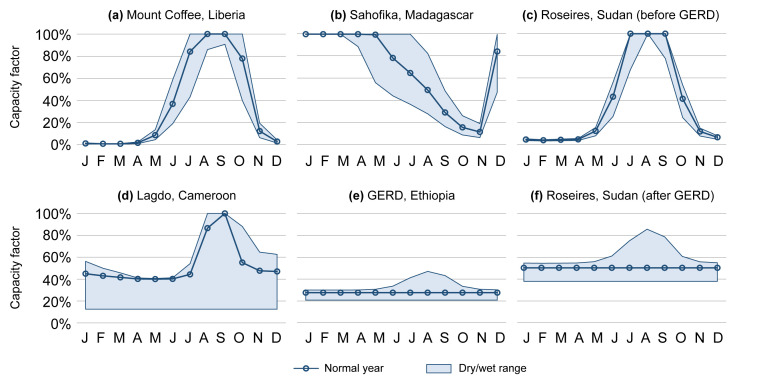
Six demonstrations of the monthly typical capacity factor profiles in the AHA (normal years as well as very dry and very wet years). Showcased are a run-of-river plant ( **a**), two reservoir plants with less-than-a-year storage capacity ( **b**– **c**), and two reservoir plants with more-than-a-year storage capacity ( **d**– **e**). Further, the plant in ( **c**) will form part of a cascade with ( **e**) in the future, resulting in profile ( **f**). GERD = Grand Ethiopian Renaissance Dam.

All above calculations were performed separately for the months of a normal, very dry, and very wet year. An example of a capacity factor profile calculated for a run-of-river hydropower plant is shown in Figure 4(a).

***2.5.2 Reservoir hydropower plants.*** For all reservoir-based plants, the reservoir inflow was separated into a “storable” and a “non-storable” component, based on the typical “filling time” of the reservoir (the time it would take for the average inflow to fill the reservoir). This approach is described in detail in the Supplementary Material of ref. 9 and briefly summarized here.

Essentially, the “storable” component corresponds to the percentage of inflow that, if cumulated across the year, would be precisely enough to fill the reservoir’s live storage volume; this component is assumed to be stored by the reservoir and redistributed equally over the different seasons (see section 3 for a discussion of this assumption). The “non-storable” component, on the other hand, corresponds to the remainder of the inflow which hence cannot be stored (as this would lead to spilling, which is to be minimized in normal reservoir operation schemes); it is therefore assumed to be directly turbined. For reservoirs with a filling time of more than one year, the non-storable component is therefore equal to zero.

In terms of volume, the “storable component” of the total yearly volume inflow thus equals the live reservoir volume, and the “non-storable component” is the part of total yearly volume inflow that exceeds this live reservoir volume. As an example, let us say a hypothetical hydropower reservoir has a (live) volume of 2, but a river carries an annual discharge volume of 8 in a “normal” year. In this case, the “storable component” of river flow is equal to 2 (25%, or 2 out of 8), and the “non-storable component” of river flow is the remainder, i.e. 6 (75%).

Note that the filling time can differ between dry and wet years; thus, a reservoir’s non-storable component may be zero during very dry years (resulting in an unseasonal outflow profile) but finite during very wet years (bringing a seasonal peak into the outflow profile) ^9^. We assumed live storage volume to be 70% of total reservoir volume in all cases.

The total outflow of the reservoir-based plants was then calculated as the sum of the redistributed “storable” and “non-storable” flow components. Subsequently, the conversion of these outflow profiles to typical monthly average capacity factor profiles was done as described by Equation (1)– Equation (3) in section 2.5.1.

The above calculation is performed independently of the initial storage level and of the demand profile. Principally, the methodology amounts to assuming that the amount of water that *can* be stored and flexibly turbined across the year without seasonality effect, *will* be stored and flexibly turbined in this way, since the idea of large reservoirs is precisely to mitigate flow seasonalities (see also the discussion in section 3). It is to be noted that seasonalities in electricity demand, although they exist, tend to be much less pronounced than those in river inflow (cf. ref. 45).

Four examples of capacity factor profiles for reservoir hydropower plants are shown in Figure 4(b)–(e), of which two with less-than-a-year (b–c) and two with more-than-a-year filling time (d–e).

***2.5.3 Cascade configurations.*** For the development of the AHA, the definition of a “cascade” was taken to refer to any one or more run-of-river plants, or plants with relatively small reservoirs, being located downstream of larger reservoir plants on the same river stretch. In such cases, the inflow profile of the first downstream run-of-river plant was taken equal to the calculated outflow profile of the upstream reservoir plant; the inflow profile of the second downstream plant was taken equal to the outflow profile of the first downstream plant; and so forth. Finally, the outflow profiles of each plant were converted to typical monthly average capacity factor profiles as described by Equation (1)– Equation (3)) in section 2.5.1.

By default, this methodology assumes that this outflow profile (as seen from the point of view of downstream run-of-river plants) does not change significantly before arriving at the downstream plant. We deem this a reasonable assumption as cascade configurations typically consist of several plants situated relatively close together geographically.

Since cascade configurations can be time-dependent – for instance, a reservoir plant may be planned or under construction upstream of an existing run-of-river plant – the outcomes of this calculation depend on the year for which the calculations are performed, and whether this is before or after the planned reservoir plant comes online. To differentiate between these cases, the AHA contains results sheets for different example years: 2020, 2030, and “All”, the former two reflecting the hydro fleets of 2020 (present-day) and 2030, respectively, and the latter reflecting the hypothetical case where all hydropower plants, including “candidate” plants, are constructed.

An example of capacity factor profiles for a hydropower plant that is currently not part of a cascade system, but will become so in the future due to upstream construction of a large reservoir plant, is provided in Figure 4(c) & (f).

***2.5.4 Data coverage.*** With these procedures, seasonal availability profiles could be calculated for 550 out of 633 hydropower plant entries in the AHA (87%). For the remaining 83 entries – mostly small existing plants for which the snapping to the simulated river network could not be performed with confidence (see section 2.4), and “candidates” with unclear locations – the profiles could not be calculated from the present version of the AHA. Future iterations of the database and the simulations may make it possible to further close this gap.

## 3. Use and limitations of AHA data in energy modelling

The data provided in the AHA is aimed at servicing the energy modelling community to enable better representation of seasonal constraints of hydropower availability at a plant-by-plant level. The best way to import these profiles into any model will depend on the specific software used.

However, the general principle of importing and applying the profiles in energy models is as follows. For run-of-river plants, the AHA profiles can be used as-is (i.e. considered fixed), as these plants are not considered to be dispatchable, and cannot ramp up or down in function, for example, of the day-night cycle of solar PV or power demand. These profiles are thus to be used in the same way as would solar PV or wind resource profiles.

For reservoir plants, the profiles denote seasonal availability constraints rather than a fixed curve of power output. Such plants can be dispatched flexibly up to a certain extent, for example, to follow demand or to aid VRE integration ^9^, constrained by typical (sub)-hourly ramping rates which are different from case to case. In such cases, the modelling should be set up in such a way as to ensure that the power plants are represented as dispatchable technologies but constrained by average seasonal availability profiles as given by the AHA.

It is important to note that the AHA represents a first attempt at providing a comprehensive, continent-wide spatiotemporal dataset for Africa. As such, it is subject to various limitations which must be considered. The most important limitations are summarised below.

First, the river flow profiles presented in this paper were obtained from simulations representing a historical period. However, potential effects of future climate change on river flow, may be substantial ^46^. A first iteration of the AHA under climate change scenarios has been included in its version 2.0 (see “Data availability”). These results were obtained by forcing SWAT+ with simulation results obtained from four bias-adjusted global climate models (GFDL-ESM2M, HadGEM2-ES, ISPL-CM5A-LR and MIROC5). The hydropower availability profiles were calculated separately for each model in the ensemble for the historical period 1975–2005 and for the future period 2075–2100, under three RCP-SSP combinations (Representative Concentration Pathways and Shared Socioeconomic Pathways, respectively): SSP1-RCP2.6, SSP4-RCP6.0, and SSP5-RCP8.5, whereby the land cover scenario is taken from the Land Use Harmonization version 2 data ^37^ and the global climate models are forced with greenhouse gas concentration trajectories associated with each RCP ^47^. We then calculated the relative difference between the results (in terms of monthly availability of hydropower plants) from the future and historical period, and imposed this relative difference on the results obtained for the historical period forced with EWEMBI (see above).

Second, the capacity factor calculations were purely based on simulated reservoir inflow and did not consider evaporation and precipitation effects on the reservoir surfaces of future reservoirs which do not form part of the hydrological network as simulated. However, the effects of this omission are expected to be relatively minor since inflow is normally by far the dominant component of reservoir water budgets. (Two notable exceptions to this rule are Lake Victoria, a natural and mostly rain-fed lake that was later dammed for hydropower generation at its outlet, and Lake Nasser, an artificial lake in the Egyptian desert whose evaporation losses are untypically substantial in comparison to the total water budget.)

Third, the calculations did not explicitly model reservoir dynamics and thus do not include the effect of seasonal hydraulic head variations on seasonal capacity factors. While this effect exists, it is typically minor except for reservoir plants with very low heads ^9^.

Fourth, the calculations took a strong supply-side view in assuming that the purpose of hydropower reservoirs is to (partly) remove the seasonality and variability of river inflow such as to stabilize power output on seasonal timescales. However, in cases where power demand itself has a strong seasonality, or in cases where other sources in the electricity mix, like solar and wind power, exhibit extremely pronounced seasonalities and these have a major effect on the supply-demand balance, reservoir hydropower may be required to follow these seasonalities rather than fully flattening the “storable” component of river flow. If the load profiles that hydropower should follow are known, corresponding calculations could be straightforwardly carried out by adapting the methodology described in section 2.5.2. However, we note that this is mostly of importance for reservoirs with more-than-a-year storage capacity (7% of entries in the AHA). For such cases, we recommend that specific case studies be undertaken on the hydropower plants in question to elucidate the potential re-introduction of seasonalities under integrated hydro-VRE operation, such as ref. 45.

Fifth, it is to be noted that our approach is statistical rather than deterministic. Instead of modeling actual reservoir dynamics from hour to hour, such as in e.g. refs. 48– 50, we used statistics of modelled time series of river flow to infer “typical” seasonal profiles of hydropower availability. The implicit assumption here is that reservoir operation may typically follow a probabilistic approach based on historical experiences.

Sixth, for all hydropower plants, there may be additional constraints not included in the AHA that impact their inclusion in energy modelling exercises. For example, there may be certain environmental outflow constraints that put further limits on monthly hydropower generation ^51^, or certain hydropower plants where power generation needs to be co-optimised with irrigation or other secondary purposes ^52^. Our simulations already account for (existing) reservoir management and irrigation practices, but given the lack of data to constrain the SWAT+ simulations on a site-by-site basis, these practices were modelled using generalised rules on such management. Despite these limitations, we note that this is the first time that an Africa-scale calibrated hydrological model has been generated and run on climate change time scales which includes such considerations of management of dams and irrigation schemes, albeit modelled in a generalised manner.

Seventh, in its current form, the AHA covers the African mainland and Madagascar. However, there is potential for small hydropower plants on other, small African island nations such as São Tomé & Príncipe and the Comoros. These are currently not covered by the hydrological simulations used for the AHA. However, these countries will be integrated into the AHA in the future, contingent upon more exhaustive river flow data becoming available.

Eighth, we note that hydrological data are here obtained via simulation with a hydrological model forced with EWEMBI model forcing data, which may introduce additional uncertainties. The EWEMBI dataset was compiled to support the bias-correction of climate input data for the impact assessments carried out in phase 2b of the Inter-Sectoral Impact Model Intercomparison Project (ISIMIP) ^47^ which contributed to the 2018 IPCC special report on the impacts of global warming of 1.5°C above pre-industrial levels and related global greenhouse gas emission pathways, and has as such been widely accepted by the scientific community. EWEMBI is based on the meteorological dataset ERA-Interim ^53^, which provides estimates of past weather conditions through numerical weather prediction models, constrained by observations from meteorological or hydrological stations, satellite data, and other sources. While uncertainties in the EWEMBI dataset are indeed larger over the African continent due to sparser observational networks as compared to elsewhere, such reanalyses are still well-constrained by the physical equations that govern weather dynamics, especially for the variable we are interested in, i.e. river discharge, which integrates precipitation over large regions and multiple weeks ^47, 54^. We also note that the EWEMBI dataset has meanwhile been superseded by the WFDE5 dataset, produced for phase 3 of ISIMIP ^55^.

Ninth, we note that ideally, all river flow profiles extracted from the SWAT+ simulations would be bias-corrected to data covering the same time period as the simulation period (cf. section 2.4), which is currently not the case. However, we note that an observational data product covering a substantial amount of the locations and a large part of the modelling period considered in this study does, to the authors’ knowledge, not exist at this point. This led the authors to consult a wide range of data sources for the bias-correction, whose time periods are not necessarily consistent with one another. Thus, it is important to note that due to the spatiotemporal limitations of the bias-correction as it was performed, this translates into related spatiotemporal inconsistencies in the quality of the AHA as well. We hope that this can be resolved in future iterations of the database.

Related to the above, we note here that other river flow datasets exist which may be used as alternative or as complement to SWAT+. Relying on a single river flow dataset may have limitations, especially for the African continent where the density of the meteorological and hydrological network is relatively low. For this reason, the authors of this paper recommend practitioners to also consider such datasets as input to AHA-type assessments to more comprehensively clarify the uncertainty associated to hydrological data and simulations. An example of a complementary dataset may be the the GloFas-ERA5 operational global river discharge reanalysis (1979-present) ^56^.

## 4. Conclusions and outlook

This paper describes a new African Hydropower Atlas, which marks the first, continent-wide spatiotemporal database of hydropower generation profiles for existing and future hydropower plants. The aim of the AHA is to provide estimates of monthly constraints on capacity factors of hydropower plants to the energy modelling community at a plant-by-plant resolution, taking the differences between moderately dry, normal, and moderately wet years into account. The data set is made freely available in a spreadsheet-based format; in the future, it may be integrated into a web-based interface to allow interactive visualization of the results and promote more widespread diffusion of the resource.

By helping energy modellers to better represent hydropower plants’ contribution to electricity mixes across Africa, the AHA may support more informed prioritisation of future hydropower projects to be developed. This is important both from a financial and an environmental point of view. On the financial side, using AHA data in energy modelling may help elucidate which hydropower plants would be most suitable to contribute to a cost-optimised configuration of future power mixes, taking into account the seasonal variability of the hydro resource. On the environmental side, we note that it is undesirable that Africa’s full hydropower potential be exploited, such that excessive ecological impacts of river-damming interventions may be avoided ^19^; using AHA data, priority could be allocated to hydropower plants whose contribution to diversified electricity mixes would be most conducive towards low costs and high VRE penetration, allowing to deprioritize and/or shelve plans for other hydropower plants and avoid lock-in to hydro-dependency ^23^.

The main contribution of this work to the existing literature is the collation of large amounts of data and their processing into a single final product. This is not to say that the data sources that have been used are necessarily the best ones available. In the future, we hope that new iterations of hydrological simulations, new knowledge on the effects of climate change, and new knowledge on existing and upcoming hydropower plants as communicated by public documents and stakeholder feedback can be integrated into the AHA to improve its quality.

## Data availability

HydroShare: Online repository of materials for an all-Africa hydropower atlas (v2.0). https://www.hydroshare.org/resource/5e8ebdc3bfd24207852539ecf219d915 ^33^.

This project contains the following underlying data:

- The AHA (v2.0) provided as a spreadsheet (.XLSX), containing the geospatial references of the hydropower plants and their technical characteristics used in the calculations, as well as their typical monthly capacity factor profiles for normal, dry and wet years
- SWAT+ simulation results used to extract river flow profiles provided as text files (.TXT). The historical runs based on EWEMBI observations are entitled “SWAT+_channel_mon_EWEMBI_hist” and “SWAT+_reservoir_mon_EWEMBI_hist”. We refer to the SWAT+ output documentation (accessible through https://swatplus.gitbook.io/docs/download-docs) for further metadata on the columns included in this .txt file.
- SWAT+ simulation results based on runs from an ensemble of global climate models (GCMs). The channel and reservoir .txt files are given in the zipped folders “SWAT+_simulations_GCM_historical.rar” and “SWAT+_simulations_GCM_ssp_rcp.rar”.
- GIS shapefile of the river sections covered in the SWAT+ simulation.

Data are available under the terms of the Creative Commons Attribution 4.0 International license (CC-BY 4.0).

The data/metadata included in the AHA spreadsheet is summarised in the table below.

**Table:** 

Parameter	Explanation	Unit
Country	The country in which the hydropower plant is located.	
Unit Name	Name of the hydropower plant as recorded.	
Status	Existing, Committed, Candidate or Planned	
Latitude	Self-explanatory	°N
Longitude	Self-explanatory	°E
River Name	The name of the river/tributary on which the hydropower plant is located	
River Basin	The name of the river basin that the hydropower plant is part of, in the widest sense of the term “river basin” (encompassing all streams whose discharge eventually drain at the same outlet at the ocean or at an endorheic lake).	
Spill From	For cascade systems : the nearest upstream plant	
River Channel ID	The SWAT+ simulation ID of the river channel on which the hydropower plant is built	
Reservoir ID	The SWAT+ simulation ID of existing reservoirs or future plants directly downstream of existing plants	
Reservoir Size	Full (live + dead) storage volume of the hydropower plant	million m ^3^
Mean Annual Discharge	Recorded mean annual discharge values at or very near the hydropower plant site used for bias- correction	m ^3^/s
Filling Time	Time it would take the mean annual inflow to completely fill the reservoir storage	days
Design Discharge	Discharge at which the hydroturbines would operate at maximum capacity (assuming constant head)	m ^3^/s
Availability	Wherever recorded: multiannual average capacity factor. Default taken to be 50%.	fraction
First Year	Year of entry into service (for future plants : assumed *earliest possibe* year)	
Capacity	Rated capacity	MW
Size Type	Small (<10 MW), Medium (<100 MW) or Large (≥100 MW)	
Shared Plant	Describes whether the electricity generated by the plant is shared between different countries.	
Shared Plant Split	In case the electricity generated by the plant is shared between different countries, this column describes whether the plant has been allocated to a single country or split across multiple countries in the database (e.g. whether it is physically located in only one or in multiple countries). In case of the latter, a separate entry is included for each of the *N* involved countries with a fraction *1/N* of the plant’s capacity allocated to each country.	
Single Source	Source in cases where a single source was consulted for all recorded data. If not applicable, the following columns (Source_status, source_location, etc.) were filled with the respective sources.	

## Code availability

Analysis code available from: https://github.com/VUB-HYDR/2021_Sterl_etal_AHA

Archived analysis code at time of publication: https://zenodo.org/record/5777129 ^57^.

License: MIT
