# Supplementary material for: A spatiotemporal atlas of hydropower in Africa for energy modelling purposes
Source: Open Res Eur. 2022 Mar 29;1:29. Originally published 2021 Mar 26. [Version 3] doi: 10.12688/openreseurope.13392.3 (PMC10445926; doi:10.12688/openreseurope.13392.3)
Supplement: Peer review history

# Peer review: Reviewer response for version 3

**DOI:** 10.21956/openreseurope.15833.r28953

**Published:** 2022-04-07

**Authors:** Jean-Philippe Vidal

**Affiliations:** 1 UR RIVERLY, INRAE, Lyon, France

The authors have in my opinion satisfactorily responded to the comments made during my first revision.

Are sufficient details of methods and materials provided to allow replication by others?

Partly

Is the rationale for creating the dataset(s) clearly described?

Yes

Are the datasets clearly presented in a useable and accessible format?

Yes

Are the protocols appropriate and is the work technically sound?

Yes

Reviewer Expertise:

Hydroclimatogy, climate variability, climate change, drought and low-flows, reservoir management

I confirm that I have read this submission and believe that I have an appropriate level of expertise to confirm that it is of an acceptable scientific standard.

# Peer review: Reviewer response for version 2

**DOI:** 10.21956/openreseurope.15526.r28473

**Published:** 2022-02-24

**Authors:** Jean-Philippe Vidal

**Affiliations:** 1 UR RIVERLY, INRAE, Lyon, France

**Overall comments**

 This data note describes an atlas of hydropower in Africa. This atlas, compiling existing databases on existing and planned hydropower plants, and continental-scale hydrological modelling, provides information on the hydropower generation profiles at all these plant/reservoir locations. It is therefore of high interest for continental-scale energy modelling. The revised version of the manuscript has been much improved following the first-round comments of reviewers, and future projections are welcome. However, there are still places in the text and specific points that lack clarity, and some methodological issues would also require clarifications and additions to the text and the database. They are detailed below.

**Main issues**

- *Section 2.3, quality of SWAT+ simulations*: The responses to comments by Benoît Hingray on this specific issues are in my view not satisfying. Hydrological modelling is in this study used to get reasonable estimates of seasonal and interannual variability of streamflow across the continent. I therefore strongly believe that an appropriate assessment of such properties is definitely required for one to be able to gain confidence in the AHA results. One may think of monthly NSE or monthly NSE with respect to the average regime, to check the ability of SWAT+ to get interannual variability right. This assessment should be included in the AHA database where available, i.e. where bias-correction has been done. More on the bias-correction below.
- *Section 2.4, “a typical range of years”*: It should be specified here what kind of years are considered here. Civil years? Hydrological years (preferably)? If hydrological years, what is the starting date and is it homogeneous over the continent? This choice is rather critical as it may have strong implications and it should be necessary justified and commented in the discussion part.
- *Section 2.4, “First, the flow profile…”*: here, in spite of modifications made after the first round of comments, I am still unclear on how the final monthly regimes of normal, dry and wet are obtained. More precisely it is not clear what is the “monthly median of the dataset”. I would tend to understand that you took the median interannual value for each month of the year and use it to build a “normal” 12-value regime. And in parallel, you considered the distribution of annual-averaged values and computed the ratio between the median value and the 5/95 percentiles to get the correction factors. And that you subsequently applied these correction factors to all 12 values of the “normal” regime. Am I right? But then the following paragraph with the scaling made me once more hesitating on what is really done. I guess a schematic of the time series processing is strongly required here readability and reproducibility purposes. The text should definitely be clear on that, as this way of taking account of inter-annual and intra-annual variability relies on strong hypotheses that would not necessarily be shared by all users.
- *Section 2.4, bias-correction*: I believe that the answers made to Benoît Hingray on this issue are only partly satisfying. Indeed, while I understand and approve the need for bias-correcting (for this specific application), there are two points that require actions on the manuscript. First, the bias-correction (scaling) is made to existing data on multi-annual mean river discharge, but nothing is said on the period used for this bias-correction, and specifically whether, for a given location, the period for comparing observed and simulated multi-annual averages is the same. This is particularly important in (some parts of) Africa where the multidecadal variability is very high and could therefore lead to unwanted bias-correction effects. Clarifications on the methodology in the text about this point are essential, and the corresponding period should be added to the database. Second, the consequences of this bias-correction for only specific locations should be discussed in the manuscript as it generates de facto possible longitudinal (but limited as I understand form the responses) but clearly spatial inconsistencies in the quality of simulations and thus in corresponding AHA products. This comment should be added to the manuscript.
- *Section 2.5.2, storable component*: I am sorry to say that in spite of my careful reading of the responses to Benoît Hingray and the modifications made to the manuscript, the intra-annual behaviour of "storable component" hypothesized here still quite unclear. Here again, as asked by the previous reviewer, a schematic would be greatly appreciated. This is all the more important for the end-users of this database, for the hypotheses (which are very strong here for this separation between storable and non-storable components) to be correctly understood and apprehended. This is all the more important that the article referred to by the authors on this point is not in Open Access, and not available to my institution (Sterl *et al*., 2020 ^1^).
- *Section 3, irrigation modelling*: I was quite surprised to learn here that the hydrological simulations take (in some way) irrigation into account while nothing is said about it in the appropriate section 2.3! Such an important information should be given (as well as appropriate references) in the description of the river flow dataset.

**Minor issues**

- Section 2.1, “multiannual average capacity factor”: the capacity factor should be clearly defined here.
- Section 2.3, “Meteorological forcing”: it should be mentioned (probably in the discussion part) that the EWEMBI dataset (Lange, 2016) used here has now been superseded by WFDE5 (Cucchi *et al*., 2020 ^2^) produced for phase 3 of ISIMIP ( https://www.isimip.org/protocol/3/).
- Table, entry “first year”: → “earliest possible year”.

Are sufficient details of methods and materials provided to allow replication by others?

Partly

Is the rationale for creating the dataset(s) clearly described?

Yes

Are the datasets clearly presented in a useable and accessible format?

Yes

Are the protocols appropriate and is the work technically sound?

Yes

Reviewer Expertise:

Hydroclimatogy, climate variability, climate change, drought and low-flows, reservoir management

I confirm that I have read this submission and believe that I have an appropriate level of expertise to confirm that it is of an acceptable scientific standard, however I have significant reservations, as outlined above.

## References

[1] : Smart renewable electricity portfolios in West Africa. *Nature Sustainability* .2020;3(9) : 10.1038/s41893-020-0539-0 710-719 10.1038/s41893-020-0539-0

[2] : WFDE5: bias-adjusted ERA5 reanalysis data for impact studies. *Earth System Science Data* .2020;12(3) : 10.5194/essd-12-2097-2020 2097-2120 10.5194/essd-12-2097-2020

# Response

**Published:** 2022-03-22

**Authors:** Sebastian Sterl

**Affiliations:** Vrije Universiteit Brussel, Belgium

*This data note describes an atlas of hydropower in Africa. This atlas, compiling existing databases on existing and planned hydropower plants, and continental-scale hydrological modelling, provides information on the hydropower generation profiles at all these plant/reservoir locations. It is therefore of high interest for continental-scale energy modelling. The revised version of the manuscript has been much improved following the first-round comments of reviewers, and future projections are welcome. However, there are still places in the text and specific points that lack clarity, and some methodological issues would also require clarifications and additions to the text and the database. They are detailed below.*

**Author Response:** We thank the reviewer for agreeing to provide feedback on our revised manuscript, and for their positive comments and constructive criticism. We provide point-by-point replies to the issues raised by the reviewer below.

*Section 2.3, quality of SWAT+ simulations: The responses to comments by Benoît Hingray on this specific issues are in my view not satisfying. Hydrological modelling is in this study used to get reasonable estimates of seasonal and interannual variability of streamflow across the continent. I therefore strongly believe that an appropriate assessment of such properties is definitely required for one to be able to gain confidence in the AHA results. One may think of monthly NSE or monthly NSE with respect to the average regime, to check the ability of SWAT+ to get interannual variability right. This assessment should be included in the AHA database where available, i.e. where bias-correction has been done. More on the bias-correction below.*

**Author Response: **We appreciate the concern of the reviewer on this point. As we mentioned in the response to the comments by Benoît Hingray, we currently have a study in preparation which evaluates the used SWAT+ modelling results for the entire African continent (Chawanda *et al*., 2022, in preparation). These results are already part of a successfully defended PhD thesis at the Vrije Universiteit Brussel (VUB) in Brussels, Belgium (thesis entitled “ *Modelling the Impact of Climate Change & Land-Use/Management Changes on African Water Resources*” by Dr. Celray James Chawanda, 2021); this chapter will become available on the university’s repository webpage in the coming months. One of the indicators for quality here is, indeed, the monthly NSE as suggested by this reviewer. Here, on an all-Africa scale, the monthly NSE is positive for roughly 60% of river sites on all-Africa scale. Once the above-mentioned study is published, we agree that the information on monthly NSE on a plant-by-plant basis could ideally be included in a future version of the AHA. We thank the reviewer for this useful suggestion. We acknowledge the fact that the cited 60% may not be considered as sufficiently high by some hydrologists, but we again would like to reiterate the fact that, as mentioned in our previous response, we find it encouraging that these 60% of positive NSE on an all-Africa scale can be obtained using the hydrological mass-balance calibration (HMBC) approach (Chawanda *et al*., 2020) which focuses on reproducing climatological mean water balance components (e.g. annual mean evaporation) and avoids the need of having to do time-series calibration. This avoids an overfitting of the model, which would yield higher NSEs but not necessarily result in a meaningful model at the continental scale.

*Section 2.4, “a typical range of years”: It should be specified here what kind of years are considered here. Civil years? Hydrological years (preferably)? If hydrological years, what is the starting date and is it homogeneous over the continent? This choice is rather critical as it may have strong implications and it should be necessary justified and commented in the discussion part.*

**Author Response: **We thank the reviewer for this observation. In fact, the sentence was poorly worded. It should have read “ *a typical range of seasonal profiles*”. This change has now been made. (The range between what we call “very dry” and “very wet” years spans is based on 5 ^th^ and 95 ^th^ percentile values of average annual flow, as the section explains, which thus represents dryness/wetness statistically occurring once every 20 years.)  

*Section 2.4, “First, the flow profile…”: here, in spite of modifications made after the first round of comments, I am still unclear on how the final monthly regimes of normal, dry and wet are obtained. More precisely it is not clear what is the “monthly median of the dataset”. I would tend to understand that you took the median interannual value for each month of the year and use it to build a “normal” 12-value regime. And in parallel, you considered the distribution of annual-averaged values and computed the ratio between the median value and the 5/95 percentiles to get the correction factors. And that you subsequently applied these correction factors to all 12 values of the “normal” regime. Am I right? But then the following paragraph with the scaling made me once more hesitating on what is really done. I guess a schematic of the time series processing is strongly required here readability and reproducibility purposes. The text should definitely be clear on that, as this way of taking account of inter-annual and intra-annual variability relies on strong hypotheses that would not necessarily be shared by all users.*

**Author Response**: The interpretation of the reviewer is 100% correct. However, we appreciate the fact that the discussion on scaling was not fully clear, so we have changed the sentence “Third, the seasonality of river flow for these three types of years (very dry, normal, and very wet, each characterized as a time series of twelve values representing the months of the year) was calculated by dividing each time series by the multiannual average flow (simple scaling).” to “Third, the seasonal river flow profile thus obtained (for very dry, normal, and very wet years), each characterised as a time series of twelve values representing the months of the year, was normalised to a dimensionless time series by dividing each time series by the simulated multiyear average flow (a single number).” Additionally, the opening sentence of the following paragraph, “Fourth, wherever possible, the three resulting time series of river inflow to each hydropower plant were additionally bias-corrected (using the simple scaling technique) to the multiannual mean river discharge value collected from existing databases and literature (see section 2.1).” was changed to “Fourth, wherever possible, the three resulting dimensionless time series of normalised river inflow to each hydropower plant were multiplied again with a bias-correction factor (simple scaling), equal to the multiannual mean river discharge value collected from existing databases and literature (again, a single number; see section 2.1).”

*Section 2.4, bias-correction: I believe that the answers made to Benoît Hingray on this issue are only partly satisfying. Indeed, while I understand and approve the need for bias-correcting (for this specific application), there are two points that require actions on the manuscript. First, the bias-correction (scaling) is made to existing data on multi-annual mean river discharge, but nothing is said on the period used for this bias-correction, and specifically whether, for a given location, the period for comparing observed and simulated multi-annual averages is the same. This is particularly important in (some parts of) Africa where the multidecadal variability is very high and could therefore lead to unwanted bias-correction effects. Clarifications on the methodology in the text about this point are essential, and the corresponding period should be added to the database. Second, the consequences of this bias-correction for only specific locations should be discussed in the manuscript as it generates de facto possible longitudinal (but limited as I understand form the responses) but clearly spatial inconsistencies in the quality of simulations and thus in corresponding AHA products. This comment should be added to the manuscript.*

**Author Response**: We appreciate this comment from the reviewer, and we agree with them. It is important to note here that a bias-correction approach that would allow (i) **all** locations in the dataset to undergo bias-correction, for (ii) the **same** time period in each case, simply does not exist, this due to the strongly varying periods for which observations are available across the African reference stations. A bias-correction for a limited subset of data points, using time periods that are not necessarily all of the same length and not necessarily consistent with the modelling period, was considered by the authors to be the “next best option”, despite clearly not being ideal. It is important to understand that the sources consulted to obtain these data were not necessarily from scientific literature. In many cases, the consulted sources were technical project documents or articles which simply state an average flow rate “as is”, without providing the specific period to which this pertains. We agree that this is not ideal, but it is also the “best we have”, in many cases. For this reason, we do not provide the “corresponding period” explicitly in the database. However, all the data sources consulted for obtaining the average flow rate for bias-correction are documented in the database. We have adapted the sentence “We note that this step is not crucial for the data product and serves mostly for refinement of final numbers, as monthly availability curves of hydropower plants could be readily estimated without performing the bias-correction step, i.e. for the remaining 40% of cases (see section 2.5.1).” in the paragraph preceding section 2.5 to “We note that this step is not crucial for the data product and serves mostly for providing a dimension to the river flow seasonality (expressing it in m ^3^/s instead of dimensionless, which allows a comparison to e.g. design discharges of hydropower plants), as the monthly availability curves of hydropower plants could still be estimated without performing the bias-correction step, i.e. for the remaining 40% of cases (see section 2.5.1).” We have also added the following paragraph to the discussion: “Ninth, we note that ideally, all river flow profiles extracted from the SWAT+ simulations would be bias-corrected to data covering the same time period as the simulation period (cf. section 2.4), which is currently not the case. However, we note that an observational data product covering a substantial amount of the locations and a large part of the modelling period considered in this study does, to the authors’ knowledge, not exist at this point. This led the authors to consult a wide range of data sources for the bias-correction, whose time periods are not necessarily consistent with one another. Thus, it is important to note that due to the spatiotemporal limitations of the bias-correction as it was performed, this translates into related spatiotemporal inconsistencies in the quality of the AHA as well. We hope that this can be resolved in future iterations of the database.”

*Section 2.5.2, storable component: I am sorry to say that in spite of my careful reading of the responses to Benoît Hingray and the modifications made to the manuscript, the intra-annual behaviour of "storable component" hypothesized here still quite unclear. Here again, as asked by the previous reviewer, a schematic would be greatly appreciated. This is all the more important for the end-users of this database, for the hypotheses (which are very strong here for this separation between storable and non-storable components) to be correctly understood and apprehended. This is all the more important that the article referred to by the authors on this point is not in Open Access, and not available to my institution (Sterl et al., 2020).*

**Author Response**: Let us say a hypothetical hydropower reservoir has a (live) volume of 2, but a river carries an annual volume of 8 in a “normal” year. In this case, the “storable component” of river flow is equal to 2 (25%, or 2 out of 8), and the “non-storable component” of river flow is the remainder, i.e. 6 (75%). We have added this example to the text to clarify this idea. We hope that this wording sufficiently clarifies this concept and that a schematic is therefore not needed. In response to the comment that the referenced article is not available to the reviewer’s institution, we are happy to report that the manual of the REVUB software, which was used in that work, contains the same discussion of “storable” and “non-storable” components, and is available in open-access on https://github.com/VUB-HYDR/REVUB/blob/master/manual/REVUB_manual.pdf (see “Note 5”).

*Section 3, irrigation modelling: I was quite surprised to learn here that the hydrological simulations take (in some way) irrigation into account while nothing is said about it in the appropriate section 2.3! Such an important information should be given (as well as appropriate references) in the description of the river flow dataset.*

**Author Response**: The implementation of existing irrigation schemes in the SWAT+ model is fully described in the reference (Chawanda et al., 2020), and section 2.3 was only meant to repeat some of the most important elements of the simulations as described in the latter paper. However, we appreciate the reviewer’s concern for clarity on the implementation of irrigation, and have added the following bullet point to the list of data sources cited in section 2.3: “- Irrigated areas were obtained from Food and Agriculture Organisation at 0.083° × 0.083° resolution (FAO; Siebert and Frenken 2014). Irrigation was implemented as explained in Chawanda et al. (2020).”

*Section 2.1, “multiannual average capacity factor”: the capacity factor should be clearly defined here.*

**Author Response**: We have added the following text: “Here, the capacity factor of any power plant is taken to refer to the electricity that it generated over a certain time period (e.g. hour, day, season, year...) divided by the power that would have been generated if the plant had run at full capacity over that entire period.”

*Section 2.3, “Meteorological forcing”: it should be mentioned (probably in the discussion part) that the EWEMBI dataset (Lange, 2016) used here has now been superseded by WFDE5 (Cucchi et al., 20202) produced for phase 3 of ISIMIP (https://www.isimip.org/protocol/3/). Thank you for this suggestion.*

**Author Response**: We have added the following text to the eighth point of the discussion: “ *We also note that the EWEMBI dataset has meanwhile been superseded by the WFDE5 dataset, produced for phase 3 of ISIMIP*” with the appropriate reference.

*Table, entry “first year”: → “earliest possible year”.*

**Author Response**: We prefer to keep it as “first year”, since it refers also to existing plants, whose first year of service is usually well-defined. However, we noticed a spelling error in the table caption (possibe à possible), which has been corrected.

# Peer review: Reviewer response for version 2

**DOI:** 10.21956/openreseurope.15526.r28223

**Published:** 2022-01-19

**Authors:** Matteo De Felice

**Affiliations:** 1 Joint Research Centre (JRC), European Commission, Petten, The Netherlands

The revised version has addressed all my previous remarks.

Are sufficient details of methods and materials provided to allow replication by others?

Yes

Is the rationale for creating the dataset(s) clearly described?

Yes

Are the datasets clearly presented in a useable and accessible format?

Yes

Are the protocols appropriate and is the work technically sound?

Yes

Reviewer Expertise:

Data science, climate analysis, energy modelling.

I confirm that I have read this submission and believe that I have an appropriate level of expertise to confirm that it is of an acceptable scientific standard.

# Peer review: Reviewer response for version 1

**DOI:** 10.21956/openreseurope.14462.r26660

**Published:** 2021-06-10

**Authors:** Benoît Hingray

**Affiliations:** 1 Grenoble-INP, IGE, Université Grenoble Alpes, Grenoble, France

Characterizing the potential for hydropower production at any location of the hydrological network is obviously key. This requires to estimate the mean resource but also its temporal variability at different temporal scales, including the seasonality and the interannual variability. The work of Sterl *et al.* aims to provide this characterisation for a large set of locations of the hydrological network in Africa, provided the catchment area of the drainage basin is large enough.

 To my knowledge, this characterisation was never proposed before. The characterization is based here on river flow time series reconstructed for a 37 years period (1980-2016) from hydrological simulations. River discharges are simulated with SWAT+ from weather pseudo-observations, that is temperature and precipitation reconstructions from different observational datasets.

 As reported by the authors in the discussion, the AHA (African Hydropower Atlas) represents a first attempt to provide a comprehensive continent wide spatio-temporal dataset for Africa. This is obviously a very relevant contribution and the AHA is expected to be an important dataset for practitioners and policy makers. A large amount of data has been also collected to characterize water reservoirs along and beside the river network.

 At the present state however, the methodology used to develop the AHA is not described in enough detail and important clarifications are needed. The AHA is also subject to important limitations. The authors list a number of those. Other important limitations are missing and have to be mentioned and perhaps also discussed. It is very likely that the dataset will be widely used in the coming years, by many “non expert” engineers and policy makers; this definitively call for a better description of the AHA and of its quality. 

 The AHA has definitively to be indexed in a short time because of its high value for engineers and policy makers but, for the different reasons mentioned here, I recommend major corrections / clarification before the indexing of the manuscript.

**Meteorological data and hydrological model**

 From what I can judge, the most critical limitation is related to the quality of hydrological reconstructions. There are here some reasons of potential concern. This has to be clearly mentioned in the discussion section (perhaps also in the abstract and in the introduction).

 Hydrological data are scarce in Africa. Hydrological data are therefore here obtained via simulation from SWAT+, a conceptual hydrological model, forced with EWEMBI weather data. EWEMBI are not observations, but estimates of past weather conditions obtained with weather observations from stations, outputs of meteorological models, satellite data (satellite data do not provide observations of precipitation but proxy)… The diversity of data used in EWEMBI have to be acknowledge in the manuscript to highlight the potential complexity of such reconstruction and the potential errors associated. The limitations of EWEMBI have also to be acknowledge. They are expected to be large in regions without or with scarce meteorological stations which is the case almost everywhere in Africa. Some reference with the relevant evaluation should be provided.

 The quality of the SWAT+ hydrological model used for the reconstruction is another possible critical point. It not presented in the manuscript and thus unknown. A section should be ideally dedicated to the description / discussion of its quality for Africa. SWAT+ has been used / evaluated for West Africa (2 references are mentioned in the paper for this). What is its performance for the rest of the continent? For the performance metrics, the authors mention the study of Chawanda *et al*. 2020, but this study only focuses on South Africa. Then, even if we just consider this region, the performance of SWAT+ is rather (very) low and I fear that many hydrologists would not consider the simulations as very useful : the NASH efficiency criterion is indeed negative for more than 35% of evaluated river sites (that is, the model is worst than a simple « constant » model, where the constant is just the « interannual mean observed discharge »).

 For South Africa, Chawanda *et al*. also mention potentially critical limitations due to the low unavailability of reservoir management data; and to the limited information on agricultural management practices. These are expected to strongly modify 1) the water balance and then the available resource and 2) the seasonality of the river discharge downstream the dams. This has likely also to be discussed, at least mentioned.

 In a conventional publication for the academic world, the reader will guess how large is the uncertainty obtained with SWAT+ simulations in this scarce data context. The AHA is however to be used by practitioners / policy makers. The limitation associated to the different datasets used / produced here have clearly to be mentioned, especially for Africa, where the density of the meteorological / hydrological network is very low. The reader can understand that there are many paths for improving the models and then the AHA dataset but I recommend that a fair evaluation of the performance of the modelling chain and of the hydrological model is presented.

 Finally, the concepts behind SWAT+ are rather simple. Other modelling approaches are possible and other have been proposed in the recent year. One important contribution here is the GloFas-ERA5 operational global river discharge reanalysis 1979-present (Harrigan *et al*. 2020) ^1^. The simulations are obtained with LISFLood a hydrological model with a long time development and evaluation worldwide. The authors should also cite this dataset and highly recommend practitioners to consider different “reanalysis” datasets to have an idea of the uncertainty / errors associated to hydrological data and simulations.

**Bias correction. **P7 C2 § 3. The principle / interest / limitation of bias correction have to be clarified.

- If bias correction is applied on simulated discharges, observations are used therefore. What is then the added value of simulations as the characterisation of hydrological regime can be done with those observations only?
- Is bias correction applied on a monthly basis (i.e. a correction function is determined for each month separately as is typically done for bias correction of climate projections)?
- If bias correction can be applied to 2 stations with observations on a same river, what is done for the stations that are in between: bias will likely occur also at these intermediate stations; if bias is not corrected there, some discontinuity / inconsistency is expected along the river network. This has likely to be commented.
- What are the reference periods in the observation and in the simulation used to calculate the bias correction factor? Are the periods the same for a given location (and then the hypothesis is made that the bias is the same at any time, for any other year of the simulation period (expected to cover a larger period than the observations?)
- The application of bias correction or not for a given location is key. It is mentioned in the metadata of the AHA ?

**Section "reservoir hydropower plants"**

 I do not understand the way the operation of reservoirs is simulated. The description given P8 section 2.5.2. is not really clear.

 The authors mention "For all reservoir-based plants, the reservoir inflow was separated into a “storable” and a “non-storable” component, based on the typical “filling time” of the reservoir (the time it would take for the average inflow to fill the reservoir). This approach is described in detail in the Supplementary Material of ref. 9 and briefly summarized here."

 I did not find this description in the SM of ref9. Note that SM is 33 pages with 10 different subsections. The "storable" term is only used once in this SM in the sub-section "Note 5 : REVUB: Reservoir simulation for small hydropower plants" dedicated to small hydropower plants. This does obvioulsy not correspond to the "large reservoir" case considered here. In the SM of ref9, the authors introduce the REVUB model to simulate the operations of reservoirs. Do they use such a simulation model here (the model REVUB is used in the subsection where the “storable” word is used)?

 If yes:

- the basics of the model should be given in the present work, at least in a SM specially dedicated to the present manuscript. The ref.9 is indeed not accessible for free, which is a critical limitation as it is mentioned as a key methodological reference for the present work.
- For the same reasons, the figures S2 and S3 of the SM of ref9 could be included in the present work for a comprehensive illustration of the REVUB model.
- The *“REVUB model simulates a baseload oriented operation strategy”* of the reservoir, i.e. the objective of the operations is the production of a baseload discharge downstream. From what I can understand, this is thus basically just a low pass filtering of the highly variable inflow in the reservoir (where the intensity of the filter is defined by the parameters in figure S3 of SM in ref9). This assumption may be not appropriate in many reservoir configurations where the operations are produced to best follow the expected (based on past year observations) annual load profile (and its multiscale temporal variability at least from sub-daily to seasonal). What is the simulation performance of such a model if used in the present work. This "performance" issue should be also likely introduced and an estimation for different reservoir contexts should be presented. The limitations of the model have then also likely to be mentioned: e.g. adequacy of the baseload production assumption, + other assumptions such as those on direct precipitation / evaporation in / from the reservoirs.

If the REVUB model is not used in the present work, the reference to the SM of ref9 is probably not more relevant and a special section should likely describe what is done there. In all cases, the "storable component" concept has to be clarified. I do not understand what is described in § 2 col 2 of p.8. How is estimated this “storable volume”; how is it used and for what then ?…

 Is the idea to consider that a filling temporal sequence is absolutely required each year? If yes, can you precise why (because of the occurrence of a dry inflow period each year that lead the reservoir level to go down to low and sub-optimal filling rates?)? Why could we not consider that the reservoir is filled all the time? This would allow for the best production efficiency (with the highest possible hydraulic head at any time)… Clarification is required here. An illustration of a given year (with the time series of the inflow, the filling rate, the outflow, the load) for a given reservoir would be welcome to understand the process and what is refered to as "storable" and "non-storable".

 The authors say "Essentially, the “storable” component corresponds to the percentage of inflow that, if cumulated across the year, would be precisely enough to fill the reservoir’s live storage volume; this component is assumed to be stored by the reservoir and redistributed equally over the different seasons (see section 3 for a discussion of this assumption)." From which initial storage level do you start to estimate this required volume? The required volume will be obviously different from one year to the other (as mentioned later in the paragraph) depending on how wet or dry the year was and on large or small the demand was in the preceding year. If you have 30 different years, you can estimate 30 different required volumes. What do you do with these different volumes?

 On the other hand, in the real world, institutions in charge of the operations of reservoirs do not know what will be the meteorological conditions for the next weeks / months : they do thus not know what will be the inflow / load demand for the next months and thus they do not know what is / will be the required volume for the current year. In this real world configuration, the institutions in charge can but use a probabilistic approach to fill the reservoir again, defining a risk level for which the reservoir will not be filled at the end of the year (in the configuration of a highly seasonal flow). So, how is estimated this "storable" component here? How do you account for this "uncertainty / predictability" issue in your approach? A deterministic approach is likely limited there. A discussion on this "operational issue" would be welcome. In all cases, one can understand that simplifications of the operation are required provided they mimic the true operations in a relevant way. In this context, the authors have also to mention the existence of studies where more realistic representations of the reservoir operation are developed (typically based on some optimisation process) (e.g. Minville *et al*. 2009 ^2^; Turner *et al*. 2017 ^3^; Danso *et al.* 2021 ^4^).

 Note: The term "storable" is to me not really appropriate if you refer to the "amount of water that is required to fill the reservoir for a given year". This required water amount is obviously smaller than the storage capacity of the reservoir. The storable water suggests : “a water amount that can be stored but this is not mandatory to store it”. And indeed, the amount of water that could be stored over a given year can be much larger than the reservoir storage capacity : in a configuration where you never have overspilling, all the inflow water volume can be stored (at a given time of the year). For me, the "non storable" component of the inflow is just the amount of water that arrives in the reservoir when the reservoir is 100% filled (something that may happen during some (limited) periods of the year).

 The authors are thus gently asked to clarify the terminology / description of the methodology here. 

**Cascade configurations**

 How was the outflow profile of upstream large reservoirs determined? This is not clear. Did you consider the output of the VURB model for the upstream reservoirs?

 As mentionned by Biemans *et al*. 2011 ^5^; reservoirs can contribute significantly to irrigation water supply in many regions and significanly modify water resource downstream. A comment on this is likely required..

**Metadata for the description of data / reservoirs**

 The description of what has been done, with what data for which reservoir / location will be key for practitioners. A table with the description of all data / metadata provided for any given location is thus probably to be given in the manuscript.

 Is there any strategy to check the metadata already collected and processed in this work? For the reservoir description (P4. §4) for instance : Are the authors in contact the "Water Resource Authorities" of the different countries to check the exhaustivity / characteristics of the dams considered in the work.

 Then it would be interesting to precise the strategy retained to update the data / metadata of the AHA. For instance to update the metadata for the description of the different "existing" and future projects? Is there any reference institution that is committed to do this update? What is the contact for this ? Do the authors expect to produce / deliver improved releases of the AHA in the coming years?

 p7 §3 : "snapping" : is the hierarchy level considered for each plant in the AHA mentioned in the metadata of the AHA (this information is obviously key)?

**Data Availability**

 I am not a typical enduser of such database but the author say that the AHA gives

 “- SWAT+ simulation results used to extract river flow profiles provided as text files (.TXT).”

 The format of the data there is surprising and makes for me the file hard to exploit. All monthly time steps of the 1980 – 2016 period are given in turn, with all 5438 hydrological units used in the SWAT+ model pooled for each time step in 5438 consecutive lines. We do not have thus access to the time series of simulated discharges for each hydrological unit individually. This could be likely improved sothat the enduser could access easily to the full time series (and not only to the yearly mean profiles) of each hydrological unit.

 I also did not find a note that describes what is contained in each file of the database. Can such a be given as a SM of the present note?

**Detailed comments**

 P4. First § : the multi-annual mean discharges. Can you precise for which period? Based on which data?

 p7. C2 - § 1 : "To account for the fact that some few hydropower plants with very large reservoirs are capable of buffering water on interannual timescales and thus mitigate interannual variability, an exception in the calculation was made for those plants with a typical filling time ^9^ of more than one full year. For these plants, instead of the 5 ^th^ and 95 ^th^ percentiles, the 10 ^th^ and 90 ^th^ percentiles were taken to account for this mitigation of dry and wet extremes on interannual timescales."

 I do understand the rationale in the previous paragraph but I would suggest that the first estimate with the 5th and 95h percentiles is given for all reservoirs and that the 10th and 90th percentiles are given as additional information for the very large reservoirs. Is it also mentioned in the AHA what is the mean time required to fill in the reservoir from the zero level?

 p7. C2 - § 2 : please reformulate "the seasonality of river flow for these three types of years (very dry, normal, and very wet, each characterized as a time series of twelve values representing the months of the year) was calculated by dividing each time series by the multiannual average flow." Did you apply simple scaling on the mean hydrological cycle characterized by 12 monthly mean values? If yes, please clarify.

 p8 C1 §3 :

 "the maximum flow" >> "the maximum monthly flow"

 "monthly profiles" >> "annual cycles"

 P10. C1 §1. “Inflow is normally by far the dominant component of reservoir water budget”. Yes, but this is not always the case, especially in arid regions. Typical % values from previous literature in different contexts should be likely mentioned there to fix ideas.

 P9 C2 – Climate change. This issue is indeed important. Some references to recent work could be mentioned (e.g. Stanzel *et al.* 2018 ^6^; Bichet *et al*. 2020 ^7^; Moussa *et al*. 2020 ^8^).

 SWAT+ : The description of SWAT+ has to be given in more details. The authors should give at least the references for the Penman-Montheith, the SCS-CN and the routing method used. To my knowledge (but upgraded versions could have been produced in the meantime), the SCS-CN method is suited for "single rainfall-runoff events" simulations, but not for "continuous" hydrological simulation covering multiple years.

Are sufficient details of methods and materials provided to allow replication by others?

Partly

Is the rationale for creating the dataset(s) clearly described?

Yes

Are the datasets clearly presented in a useable and accessible format?

Partly

Are the protocols appropriate and is the work technically sound?

Yes

Reviewer Expertise:

Hydroclimatology, Hydropower, Mutlipurpose Water Reservoir management and modelling, Climate change impacts and regionalisation, Energy transition, Renewable Energy.

I confirm that I have read this submission and believe that I have an appropriate level of expertise to confirm that it is of an acceptable scientific standard, however I have significant reservations, as outlined above.

## References

[1] : GloFAS-ERA5 operational global river discharge reanalysis 1979–present. *Earth System Science Data* .2020;12(3) : 10.5194/essd-12-2043-2020 2043-2060 10.5194/essd-12-2043-2020

[2] : Adaptation to Climate Change in the Management of a Canadian Water-Resources System Exploited for Hydropower. *Water Resources Management* .2009;23(14) : 10.1007/s11269-009-9418-1 2965-2986 10.1007/s11269-009-9418-1

[3] : Assessing hydropower flexibility for integrating solar and wind energy in West Africa using dynamic programming and sensitivity analysis. Illustration with the Akosombo reservoir, Ghana. *Journal of Cleaner Production* .2021;287: 10.1016/j.jclepro.2020.125559 10.1016/j.jclepro.2020.125559

[4] : Examining global electricity supply vulnerability to climate change using a high-fidelity hydropower dam model. *Sci Total Environ* .2017;590-591: 10.1016/j.scitotenv.2017.03.022 663-675 10.1016/j.scitotenv.2017.03.022 28283290

[5] : Impact of reservoirs on river discharge and irrigation water supply during the 20th century. *Water Resources Research* .2011;47(3) : 10.1029/2009WR008929 10.1029/2009WR008929

[6] : Climate change impact on West African rivers under an ensemble of CORDEX climate projections. *Climate Services* .2018;11: 10.1016/j.cliser.2018.05.003 36-48 10.1016/j.cliser.2018.05.003

[7] : Assessing uncertainties in the regional projections of precipitation in CORDEX-AFRICA. *Climatic Change* .2020;162(2) : 10.1007/s10584-020-02833-z 583-601 10.1007/s10584-020-02833-z

[8] : Near-term impacts of climate variability and change on hydrological systems in West and Central Africa. *Climate Dynamics* .2020;54(3-4) : 10.1007/s00382-019-05102-7 2041-2070 10.1007/s00382-019-05102-7

# Response

**Published:** 2021-12-13

**Authors:** Sebastian Sterl

**Affiliations:** Vrije Universiteit Brussel, Belgium

(Author Responses in italics)

 Characterizing the potential for hydropower production at any location of the hydrological network is obviously key. This requires to estimate the mean resource but also its temporal variability at different temporal scales, including the seasonality and the interannual variability. The work of Sterl et al. aims to provide this characterisation for a large set of locations of the hydrological network in Africa, provided the catchment area of the drainage basin is large enough. To my knowledge, this characterisation was never proposed before. The characterization is based here on river flow time series reconstructed for a 37 years period (1980-2016) from hydrological simulations. River discharges are simulated with SWAT+ from weather pseudo-observations, that is temperature and precipitation reconstructions from different observational datasets. As reported by the authors in the discussion, the AHA (African Hydropower Atlas) represents a first attempt to provide a comprehensive continent wide spatio-temporal dataset for Africa. This is obviously a very relevant contribution and the AHA is expected to be an important dataset for practitioners and policy makers. A large amount of data has been also collected to characterize water reservoirs along and beside the river network. At the present state however, the methodology used to develop the AHA is not described in enough detail and important clarifications are needed. The AHA is also subject to important limitations. The authors list a number of those. Other important limitations are missing and have to be mentioned and perhaps also discussed. It is very likely that the dataset will be widely used in the coming years, by many “non expert” engineers and policy makers; this definitively call for a better description of the AHA and of its quality. The AHA has definitively to be indexed in a short time because of its high value for engineers and policy makers but, for the different reasons mentioned here, I recommend major corrections / clarification before the indexing of the manuscript.

***Author Response:*** *We thank the reviewer for their positive words about our manuscript, and are grateful for the constructive and very extensive criticism which we have done our best to take into account.*

**Meteorological data and hydrological model** From what I can judge, the most critical limitation is related to the quality of hydrological reconstructions. There are here some reasons of potential concern. This has to be clearly mentioned in the discussion section (perhaps also in the abstract and in the introduction). Hydrological data are scarce in Africa. Hydrological data are therefore here obtained via simulation from SWAT+, a conceptual hydrological model, forced with EWEMBI weather data. EWEMBI are not observations, but estimates of past weather conditions obtained with weather observations from stations, outputs of meteorological models, satellite data (satellite data do not provide observations of precipitation but proxy)… The diversity of data used in EWEMBI have to be acknowledge in the manuscript to highlight the potential complexity of such reconstruction and the potential errors associated. The limitations of EWEMBI have also to be acknowledged. They are expected to be large in regions without or with scarce meteorological stations which is the case almost everywhere in Africa. Some reference with the relevant evaluation should be provided.

***Author Response:*** *We thank the reviewer for this comment. We have added an eighth point of discussion in the “Discussion” section, which acknowledges the diversity of data of EWEMBI but notes also the wide scientific acceptance of the EWEMBI dataset. We note here also a few additional points as to why we consider the EWEMBI dataset to be the go-to dataset for this undertaking, specifically the fact that (i) despite the relative scarcity of observational stations on the African continent, reanalyses are well-constrained by the physical equations that govern weather dynamics, and (ii) the variables we are interested in integrate precipitation data over large regions and multiple weeks.*

 The quality of the SWAT+ hydrological model used for the reconstruction is another possible critical point. It not presented in the manuscript and thus unknown. A section should be ideally dedicated to the description / discussion of its quality for Africa. SWAT+ has been used / evaluated for West Africa (2 references are mentioned in the paper for this). What is its performance for the rest of the continent? For the performance metrics, the authors mention the study of Chawanda *et al.* 2020, but this study only focuses on South Africa. Then, even if we just consider this region, the performance of SWAT+ is rather (very) low and I fear that many hydrologists would not consider the simulations as very useful : the NASH efficiency criterion is indeed negative for more than 35% of evaluated river sites (that is, the model is worst than a simple « constant » model, where the constant is just the « interannual mean observed discharge »).

***Author Response:*** *We thank the reviewer for this comment.* *In classical hydrology, one would use observational time series for calibration. For computational reasons, this approach is not feasible on continental-scale applications such as the AHA. This is why we developed hydrological mass-balance calibration (HSMBC, see Chawanda et al. 2020), which focuses on reproducing long-term mean water balance terms (e.g., evapotranspiration, baseflow) and which is showcased for South Africa in the cited paper. Even though this may give lower quality (expressed e.g. with the NASH efficiency criterion) than a time-series calibration, it has the major advantage that it is applicable on continental scales without overfitting the hydrological model everywhere and at the same time allowing for considerable computational benefits.* *Given this comparatively simple approach to hydrological model calibration, we consider it encouraging that the NASH efficiency criterion here is positive for roughly 60% of river sites on all-Africa scale. We currently have a study in preparation which evaluates and applies this method to the whole of Africa (Chawanda et al., in prep.). In addition, for the specific purposes of the AHA, we note that we performed an additional bias-correction of river discharge at hydropower / reservoir sites based on observational time series, as described in the paper (section 2.4).*

 For South Africa, Chawanda *et al.* also mention potentially critical limitations due to the low unavailability of reservoir management data; and to the limited information on agricultural management practices. These are expected to strongly modify 1) the water balance and then the available resource and 2) the seasonality of the river discharge downstream the dams. This has likely also to be discussed, at least mentioned.

***Author Response:*** *Thank you for this suggestion. Our SWAT+ simulations currently account for (existing) reservoir management and irrigation practices in a simplified way. Given the lack of data to constrain the SWAT+ simulations on a site-by-site basis, these practices were modelled using generalised rules on such management. Despite these limitations, we note that this is the first time that an Africa-scale calibrated hydrological model has been generated and run on climate change time scales which includes such considerations of management of dams and irrigation schemes, albeit modelled in a generalised manner. More details are provided in Chawanda et al. 2020. We have included this explanation in section 3 of the revised paper.*

 In a conventional publication for the academic world, the reader will guess how large is the uncertainty obtained with SWAT+ simulations in this scarce data context. The AHA is however to be used by practitioners / policy makers. The limitation associated to the different datasets used / produced here have clearly to be mentioned, especially for Africa, where the density of the meteorological / hydrological network is very low. The reader can understand that there are many paths for improving the models and then the AHA dataset but I recommend that a fair evaluation of the performance of the modelling chain and of the hydrological model is presented. Finally, the concepts behind SWAT+ are rather simple. Other modelling approaches are possible and other have been proposed in the recent year. One important contribution here is the GloFas-ERA5 operational global river discharge reanalysis 1979-present (Harrigan et al. 2020)1. The simulations are obtained with LISFLood a hydrological model with a long time development and evaluation worldwide. The authors should also cite this dataset and highly recommend practitioners to consider different “reanalysis” datasets to have an idea of the uncertainty / errors associated to hydrological data and simulations.

***Author Response:*** *We thank the reviewer for this useful addition. In the revised version of the manuscript, we now cite the proposed datasets as potential alternatives to the SWAT+ dataset used in the present study, including the recommendation to undertake similar analyses in the future based on these datasets for a more comprehensive uncertainty assessment and the rationale behind this (low density of met/hydro station network on the African continent). We also note that an upcoming publication (Chawanda et al., in prep.) analyses the SWAT+ results in details and includes an uncertainty assessment. While too late to be cited in the current AHA study, we hope that this information will be available to the scientific community in due course.*

**Bias correction. **P7 C2 § 3. The principle / interest / limitation of bias correction have to be clarified. If bias correction is applied on simulated discharges, observations are used therefore. What is then the added value of simulations as the characterisation of hydrological regime can be done with those observations only?

***Author Response:*** *The added value of the simulations is to provide data on (i) seasonality and (ii) interannual variability of river flow, since the bias correction is based only on observed/cited long-term mean discharges. Clearly, one needs observations on seasonalities and interannual variabilities to infer long-term mean discharges, but the simple fact is that the latter are widely available in literature and public datasets, whereas the former are usually not. We note here that our methodology, as explained in the paper, is also able of estimating typical capacity factor profiles on seasonal level even for hydropower plants where no long-term mean discharges are available to bias-correct the simulated data. Effectively, the bias-correction is just a “final step”, wherever possible, to refine somewhat the simulated patterns.*  

 Is bias correction applied on a monthly basis (i.e. a correction function is determined for each month separately as is typically done for bias correction of climate projections)?

***Author Response:*** *Please see our response to the above comment. Bias-correction is done on the basis of multi-annual mean discharges (see p.7, right column, third section). Monthly river flow time series are not publicly available to an extent that would have permitted monthly basis bias-correction (note, in this context, that we attempted to rely solely on publicly available information to safeguard the open-access character of the hydropower atlas, so we did not consider restricted or even commercial acquisition of river flow data to be an option).*

 If bias correction can be applied to 2 stations with observations on a same river, what is done for the stations that are in between: bias will likely occur also at these intermediate stations; if bias is not corrected there, some discontinuity / inconsistency is expected along the river network. This has likely to be commented.

***Author Response:*** *Bias-correction was only performed for cases where either (i) mean discharge values were available for the hydropower site in question (e.g. from environmental impact assessments where measurements directly at the hydropower plant construction site were invoked), or (ii) mean discharge values were available from gauging stations located very close by. We realise that “very close by” contains a degree of subjectivity, but in practice, it means that for cases cited by the reviewer, bias-correction was abstained from for such “intermediate stations” since no accurate measurements on long-term mean discharge would have been available.* *A comment on this has been included in the manuscript (end of section 2.4).*

 What are the reference periods in the observation and in the simulation used to calculate the bias correction factor? Are the periods the same for a given location (and then the hypothesis is made that the bias is the same at any time, for any other year of the simulation period (expected to cover a larger period than the observations?)

***Author Response:*** *We did not work with a specific reference period for the bias-correction, since it is practically impossible to find data on multiannual mean discharge covering exactly the same period for the hundreds of locations surveyed. The reference period of the simulation is 1980-2016 (37 years) and the bias-correction factors (which should be seen more as an estimation, to be in the right order of magnitude as compared to the plant's design discharge, than exact measures of mean discharge) may be from within this period or outside it, depending on the data source that was consulted for each.* *We have clarified in the paper that the bias-correction is meant rather as an estimation to be compared to design discharge than as a highly accurate measure of absolute flow rates (end of section 2.4).*

 The application of bias correction or not for a given location is key. It is mentioned in the metadata of the AHA?

***Author Response:*** *Yes. Wherever a multiannual mean discharge value was available as the basis for bias-correction, it is recorded in a separate column in the AHA.*

**Section "reservoir hydropower plants"** I do not understand the way the operation of reservoirs is simulated. The description given P8 section 2.5.2. is not really clear. The authors mention "For all reservoir-based plants, the reservoir inflow was separated into a “storable” and a “non-storable” component, based on the typical “filling time” of the reservoir (the time it would take for the average inflow to fill the reservoir). This approach is described in detail in the Supplementary Material of ref. 9 and briefly summarized here." I did not find this description in the SM of ref9. Note that SM is 33 pages with 10 different subsections. The "storable" term is only used once in this SM in the sub-section "Note 5 : REVUB: Reservoir simulation for small hydropower plants" dedicated to small hydropower plants. This does obviously not correspond to the "large reservoir" case considered here. In the SM of ref9, the authors introduce the REVUB model to simulate the operations of reservoirs. Do they use such a simulation model here (the model REVUB is used in the subsection where the “storable” word is used)?

***Author Response:*** *The REVUB model was not used in the present paper. It is indeed the sub-section to which the reviewer refers that contains the relevant information. Note that “small” in the referenced SM means “having less-than-a-year storage capacity”. In this regard, the “storable” and “non-storable” components indeed are not relevant for large reservoirs (defined as having “more-than-a-year storage capacity”), where all inflow is “storable”. Please see also our responses further below.*

 If yes: the basics of the model should be given in the present work, at least in a SM specially dedicated to the present manuscript. The ref.9 is indeed not accessible for free, which is a critical limitation as it is mentioned as a key methodological reference for the present work. For the same reasons, the figures S2 and S3 of the SM of ref9 could be included in the present work for a comprehensive illustration of the REVUB model. The “REVUB model simulates a baseload oriented operation strategy” of the reservoir, i.e. the objective of the operations is the production of a baseload discharge downstream. From what I can understand, this is thus basically just a low pass filtering of the highly variable inflow in the reservoir (where the intensity of the filter is defined by the parameters in figure S3 of SM in ref9). This assumption may be not appropriate in many reservoir configurations where the operations are produced to best follow the expected (based on past year observations) annual load profile (and its multiscale temporal variability at least from sub-daily to seasonal). What is the simulation performance of such a model if used in the present work. This "performance" issue should be also likely introduced and an estimation for different reservoir contexts should be presented. The limitations of the model have then also likely to be mentioned: e.g. adequacy of the baseload production assumption, + other assumptions such as those on direct precipitation / evaporation in / from the reservoirs.

***Author Response:*** *Since the REVUB model is not used in the present work, we refer to the other responses.*

 If the REVUB model is not used in the present work, the reference to the SM of ref9 is probably not more relevant and a special section should likely describe what is done there. In all cases, the "storable component" concept has to be clarified. I do not understand what is described in § 2 col 2 of p.8. How is estimated this “storable volume”; how is it used and for what then ?…

***Author Response:*** *The storable volume represents simply the volume of the hydropower reservoir, minus the “dead storage” component (that is, the volume that can be refilled and drawn down each year). For hydropower plants with less-than-a-year storage capacity, the total yearly volume inflow exceeds this storable volume. The “storable component” of the total yearly volume inflow thus equals the live reservoir volume, and the “non-storable component” is the part of total yearly volume inflow that exceeds this reservoir volume.* *We have made the language somewhat clearer in order to prevent confusion with the reader (section 2.5.2).*  

 Is the idea to consider that a filling temporal sequence is absolutely required each year? If yes, can you precise why (because of the occurrence of a dry inflow period each year that lead the reservoir level to go down to low and sub-optimal filling rates?)? Why could we not consider that the reservoir is filled all the time? This would allow for the best production efficiency (with the highest possible hydraulic head at any time)… Clarification is required here. An illustration of a given year (with the time series of the inflow, the filling rate, the outflow, the load) for a given reservoir would be welcome to understand the process and what is referred to as "storable" and "non-storable".

***Author Response:*** *Indeed a filling sequence is required in many cases and is typical in practice. Many rivers across the African continent are highly seasonal in character. The purpose of constructing reservoirs is to allow to flatten out these seasonalities (since they are typically much stronger than the seasonalities in electricity demand) by storing some of the incoming water in the wet season and using it to produce electricity in the dry season.* *For highly seasonal rivers with pronounced dry and wet seasons, insisting that a reservoir have stable water levels throughout the year basically would amount to filling the reservoir once (after commissioning of the plant) and subsequently always turbining the water as it comes in (e.g. turbining almost nothing in the dry season, turbining a lot in the wet season). This would mean that plant operation would be basically imitating a run-of-river plant, which would serve no general purpose since then one could simply have left out the reservoir. This is more important than ensuring that the hydraulic head is always constant (because what good is hydraulic head if you can't turbine water anyway during the dry season?).* *We note here that the calculations performed are estimations of the seasonal character of hydropower availability, fully reproducible according to equations 1-3 and the explanations in 2.5.1-2.5.2. These equations do not “step forward in time” without knowing the future. Rather, they use already available time series of reservoir inflow to estimate monthly turbined flow and converting the latter to capacity factors.* *The concepts of “storable” and “non-storable” have been elaborated in the revision (section 2.5.2).* 

 The authors say "Essentially, the “storable” component corresponds to the percentage of inflow that, if cumulated across the year, would be precisely enough to fill the reservoir’s live storage volume; this component is assumed to be stored by the reservoir and redistributed equally over the different seasons (see section 3 for a discussion of this assumption)." From which initial storage level do you start to estimate this required volume? The required volume will be obviously different from one year to the other (as mentioned later in the paragraph) depending on how wet or dry the year was and on large or small the demand was in the preceding year. If you have 30 different years, you can estimate 30 different required volumes. What do you do with these different volumes?

***Author Response:*** This calculation is performed independently of the initial storage level and of the demand. Basically, our methodology amounts to assuming that the amount of water that *can* be stored and flexibly turbined across the year is roughly equal to the live storage volume of the reservoir (we deem this a reasonable assumption, since this is what is physically possible, and the idea should be to flatten the seasonality as much as possible, in the sense that demand seasonality is usually much smaller than river flow seasonality, see e.g. Sterl et al. 2021: *https://www.nature.com/articles/s41560-021-00799-5* *). If one had not wished to flatten seasonality to the extent implied by this methodology, one could have conceived a smaller reservoir and inundated a smaller area.* *We have included a comment in the manuscript that summarises the above (section 2.5.2).* *Insofar as demand is concerned, this issue is already addressed on p. 10, left column, S3.*

 On the other hand, in the real world, institutions in charge of the operations of reservoirs do not know what will be the meteorological conditions for the next weeks / months : they do thus not know what will be the inflow / load demand for the next months and thus they do not know what is / will be the required volume for the current year. In this real world configuration, the institutions in charge can but use a probabilistic approach to fill the reservoir again, defining a risk level for which the reservoir will not be filled at the end of the year (in the configuration of a highly seasonal flow). So, how is estimated this "storable" component here? How do you account for this "uncertainty / predictability" issue in your approach? A deterministic approach is likely limited there. A discussion on this "operational issue" would be welcome. In all cases, one can understand that simplifications of the operation are required provided they mimic the true operations in a relevant way. In this context, the authors have also to mention the existence of studies where more realistic representations of the reservoir operation are developed (typically based on some optimisation process) (e.g. Minville *et al.* 2009; Turner et al. 2017; Danso *et al*. 2021).

***Author Response:*** *We thank the reviewer for this comment. It is to be noted that our approach is statistical rather than actually deterministic – we do not model actual reservoir dynamics from hour to hour; rather, we use statistics (medians, percentile ranges) of modelled time series of river flow to infer “typical” profiles of hydropower generation. The implicit assumption here is indeed that reservoir operation follows a probabilistic approach based on historical experiences. We have added a comment in the discussion section (3) to clarify this point, and also cited some of the literature cited by the reviewer.*

 Note: The term "storable" is to me not really appropriate if you refer to the "amount of water that is required to fill the reservoir for a given year". This required water amount is obviously smaller than the storage capacity of the reservoir. The storable water suggests : “a water amount that can be stored but this is not mandatory to store it”. And indeed, the amount of water that could be stored over a given year can be much larger than the reservoir storage capacity : in a configuration where you never have overspilling, all the inflow water volume can be stored (at a given time of the year). For me, the "non storable" component of the inflow is just the amount of water that arrives in the reservoir when the reservoir is 100% filled (something that may happen during some (limited) periods of the year). The authors are thus gently asked to clarify the terminology / description of the methodology here.

***Author Response:*** *We believe that this question has already been answered in our response to the previous comments and the issue clarified in the new version of the manuscript.*

**Cascade configurations** How was the outflow profile of upstream large reservoirs determined? This is not clear. Did you consider the output of the VURB model for the upstream reservoirs? The outflow profiles of reservoir plants (whether large or small) were determined as described in section 2.5.2, assuming (as mentioned in the comments above) that the full range of reservoir live storage is used to flatten out seasonality as much as possible by redistributing the storable component of river inflow equally across the months, and subsequently adding the non-storable component (whose seasonality is retained).

***Author Response:*** *It is true that this methodology assumes that this outflow profile (as seen from the point of view of downstream run-of-river plants) does not change significantly before arriving at the downstream plant. We deem this a reasonable assumption as cascade configurations typically consist of several plants situated relatively close together geographically, but this was not clear in the previous version of the manuscript. We have included a comment in the manuscript to clarify this point (section 2.5.3).*  

 As mentioned by Biemans et al. 2011; reservoirs can contribute significantly to irrigation water supply in many regions and significantly modify water resource downstream. A comment on this is likely required.

***Author Response:*** *A comment on the issue of irrigation water requirements is already mentioned in the discussion section: “there may be (…) certain hydropower plants where power generation needs to be co-optimised with irrigation”.* 

**Metadata for the description of data / reservoirs** The description of what has been done, with what data for which reservoir / location will be key for practitioners. A table with the description of all data / metadata provided for any given location is thus probably to be given in the manuscript.

***Author Response:*** *We thank the reviewer for this observation. A table with all data / metadata provided for any location is now given in the manuscript (section “data availability”).*

 Is there any strategy to check the metadata already collected and processed in this work? For the reservoir description (P4. §4) for instance : Are the authors in contact the "Water Resource Authorities" of the different countries to check the exhaustivity / characteristics of the dams considered in the work.

***Author Response:*** *Unfortunately, this is not yet the case, as it fell outside of temporal and budgetary possibilities available to the authors. We are interested in pursuing such a strategy in the future contingent upon budgetary possibilities.*

 Then it would be interesting to precise the strategy retained to update the data / metadata of the AHA. For instance to update the metadata for the description of the different "existing" and future projects? Is there any reference institution that is committed to do this update? What is the contact for this ? Do the authors expect to produce / deliver improved releases of the AHA in the coming years?

***Author Response:*** *Yes, it is expected that improved releases of the AHA will be published in the coming years as new information gets included in the database. The institution committed to do this update is the International Renewable Energy Agency (IRENA) in collaboration with the Vrije Universiteit Brussel. The contact, at this moment, is the lead author of this paper.*  

 p7 §3 : "snapping" : is the hierarchy level considered for each plant in the AHA mentioned in the metadata of the AHA (this information is obviously key)? *Yes, the AHA includes a column “source location” which describes the source of the coordinates as included.*

**Data Availability** I am not a typical end-user of such database but the author say that the AHA gives “- SWAT+ simulation results used to extract river flow profiles provided as text files (.TXT).” The format of the data there is surprising and makes for me the file hard to exploit. All monthly time steps of the 1980 – 2016 period are given in turn, with all 5438 hydrological units used in the SWAT+ model pooled for each time step in 5438 consecutive lines. We do not have thus access to the time series of simulated discharges for each hydrological unit individually. This could be likely improved so that the end-user could access easily to the full time series (and not only to the yearly mean profiles) of each hydrological unit.

***Author Response:*** *This is the default output format provided by the SWAT+ simulations, but it is straightforward to regroup the data to obtain the simulated discharges for each unit individually. In fact, the Python code written to analyse the data does precisely this before moving on to the other elements of the analysis, and this code has been provided open-source along with the paper. It should thus be straightforward for a potential data user to obtain the time series cited by the reviewer (the variable “flow_extract” in the code).* *We refer to the SWAT+ output documentation (accessible through* *https://swatplus.gitbook.io/docs/download-docs* *) for further metadata on the columns included in the SWAT+ output .txt file.*

 I also did not find a note that describes what is contained in each file of the database. Can such a be given as a SM of the present note?

***Author Response:*** *In case the reviewer refers to the SWAT+ results when referring to the database, this information is given in the section “Data Availability”. In case the reviewer refers to the AHA itself, this information is provided on the first worksheet “0 - Overview” of the spreadsheet file available on HydroShare.*

**Detailed comments** P4. First § : the multi-annual mean discharges. Can you precise for which period? Based on which data?

***Author Response:*** *See our comment above (fourth response in the section “bias correction”).*

 p7. C2 - § 1 : "To account for the fact that some few hydropower plants with very large reservoirs are capable of buffering water on interannual timescales and thus mitigate interannual variability, an exception in the calculation was made for those plants with a typical filling time9 of more than one full year. For these plants, instead of the 5th and 95th percentiles, the 10th and 90th percentiles were taken to account for this mitigation of dry and wet extremes on interannual timescales." I do understand the rationale in the previous paragraph but I would suggest that the first estimate with the 5th and 95h percentiles is given for all reservoirs and that the 10th and 90th percentiles are given as additional information for the very large reservoirs. Is it also mentioned in the AHA what is the mean time required to fill in the reservoir from the zero level?

***Author Response:*** *We thank the reviewer for this comment. While we see the rationale of the reviewer’s suggestion, we prefer to leave the database as it is without adding additional percentile ranges, as this is likely to lead to confusion as to what our data suggests is representative of “very dry” and “very wet” years. However, it should be noted that, since the code and the AHA are open-access and open-source, any interested reader could re-run the code and extract the 5th and 95th percentiles for all plants. The only necessary adaptation would be changing *range_pct_dry_res *and  *range_pct_wet_res *to the desired values in the code “load_flows.py” (e.g. to 5 and 95, instead of 10 and 90).*

 p7. C2 - § 2 : please reformulate "the seasonality of river flow for these three types of years (very dry, normal, and very wet, each characterized as a time series of twelve values representing the months of the year) was calculated by dividing each time series by the multiannual average flow." Did you apply simple scaling on the mean hydrological cycle characterized by 12 monthly mean values? If yes, please clarify.

***Author Response:*** *Correct. Basically, each of 12 monthly mean values (separately for dry, normal and wet years) was divided by the mean multiannual flow, such as to obtain a “normalised seasonality” including the information of whether a year is dry or wet compared to the long-term average. We have clarified this in the manuscript by adding a note that this refers to a simple scaling.*

 p8 C1 §3 : "the maximum flow" >> "the maximum monthly flow" "monthly profiles" >> "annual cycles" *Corrected with thanks.*   P10. C1 §1. “Inflow is normally by far the dominant component of reservoir water budget”.

 Yes, but this is not always the case, especially in arid regions. Typical % values from previous literature in different contexts should be likely mentioned there to fix ideas.

***Author Response:*** *We thank the reviewer for this comment and have added a sentence in the discussion to clarify this.* 

 P9 C2 – Climate change. This issue is indeed important. Some references to recent work could be mentioned (e.g. Stanzel *et al*. 2018; Bichet *et al.* 2020; Moussa *et al.* 2020).

***Author Response:*** *We fully agree that this issue is important. During the revision of the paper, we developed the next version of the AHA (version 2.0), which now includes a first iteration of climate change scenario results. Dedicated SWAT+ simulations were performed under three RCP-SSP combinations and the AHA results were recalculated based on this. Thus, the AHA v2.0 contains now three extra sheets for these scenarios. We hope that this will add much additional value to the dataset and be to the reviewer’s satisfaction. The following text was added to the revised paper: “A first iteration of the AHA under climate change scenarios has been included in its version 2.0 (see “Data availability”). These results were obtained by forcing SWAT+ with simulation results obtained from four bias-adjusted global climate models (GFDL-ESM2M, HadGEM2-ES, ISPL-CM5A-LR and MIROC5). The hydropower availability profiles were calculated separately for each model in the ensemble for the historical period 1975-2005 and for the future period 2075-2100, under three RCP-SSP combinations (Representative Concentration Pathways and Shared Socioeconomic Pathways, respectively): SSP1-RCP2.6, SSP4-RCP6.0, and SSP5-RCP8.5, whereby the land cover scenario is taken from the Land Use Harmonization version 2 data (Hurtt et al., 2020) and the global climate models are forced with greenhouse gas concentration trajectories associated with each RCP (Frieler et al., 2017). We then calculated the relative difference between the results (in terms of monthly availability of hydropower plants) from the future and historical period, and imposed this relative difference on the results obtained for the historical period forced with EWEMBI (see above).”*

 SWAT+ : The description of SWAT+ has to be given in more details. The authors should give at least the references for the Penman-Montheith, the SCS-CN and the routing method used. To my knowledge (but upgraded versions could have been produced in the meantime), the SCS-CN method is suited for "single rainfall-runoff events" simulations, but not for "continuous" hydrological simulation covering multiple years.

***Author Response:*** *We thank the reviewer for this comment. We have included references for the Penman-Monteith, SCS-CN and variable storage routing methods.* *To the authors’ knowledge, continuous hydrological simulations using the SCS-CN method are well-documented in literature (e.g. Cho et al. 2018,* *https://onlinelibrary.wiley.com/doi/abs/10.1002/hyp.11463* *;  Geetha et al. 2008,* *https://link.springer.com/article/10.1007/s11269-006-9149-5* *).*

# Peer review: Reviewer response for version 1

**DOI:** 10.21956/openreseurope.14462.r26658

**Published:** 2021-04-09

**Authors:** Matteo De Felice

**Affiliations:** 1 Joint Research Centre (JRC), European Commission, Petten, The Netherlands

This article gives a clear description of the African Hydropower Atlas (AHA).

 The authors explain all the steps behind the creation of the data, and in general the methodology is very clear. The task of creating a complete and accurate dataset of all current and future hydropower projects in Africa is definitely challenging and I can say that the authors succeeded in this task.

 However, I would like to mention a few points that can be addressed or discussed to improve the work:

1. There are some hydropower plants that are built as a joint initiative of multiple countries which agree then to share the electricity generated. A clear example is the Manantali plant which is actually in Mali but its electricity is shared with Senegal (33%) and Mauritania (15%). The authors should consider to mention this and the possibility to add this type of information in the future releases because it can be very relevant for countries with a minimal electrical infrastructure.
2. Do the authors have tried to validate the seasonal patterns of the capacity factors? For example, some Transmission System Operators in African countries publish information on their hydropower plants.
3. The data published is very rich but the text files lack metadata. The authors should add: a) information on the meaning of the columns in the txt files and b) information on the linkage between the Excel file and the SWAT data, in other words what the link between items in SWAT_reservoir_mon_EWEMBI_hist.txt and the plants in the Excel file?

I think the third point is very important to make the data published more usable and accessible. I would also invite the authors of using a more user-friendly format for their data in the future, for example considering creating a Tabular Data Package.

Are sufficient details of methods and materials provided to allow replication by others?

Yes

Is the rationale for creating the dataset(s) clearly described?

Yes

Are the datasets clearly presented in a useable and accessible format?

Yes

Are the protocols appropriate and is the work technically sound?

Yes

Reviewer Expertise:

Data science, climate analysis, energy modelling.

I confirm that I have read this submission and believe that I have an appropriate level of expertise to confirm that it is of an acceptable scientific standard.

# Response

**Published:** 2021-12-13

**Authors:** Sebastian Sterl

**Affiliations:** Vrije Universiteit Brussel, Belgium

This article gives a clear description of the African Hydropower Atlas (AHA). The authors explain all the steps behind the creation of the data, and in general the methodology is very clear. The task of creating a complete and accurate dataset of all current and future hydropower projects in Africa is definitely challenging and I can say that the authors succeeded in this task. *We thank the reviewer for their positive words about our manuscript and are grateful for the constructive criticism which we have done our best to take into account.*   However, I would like to mention a few points that can be addressed or discussed to improve the work:

- There are some hydropower plants that are built as a joint initiative of multiple countries which agree then to share the electricity generated. A clear example is the Manantali plant which is actually in Mali but its electricity is shared with Senegal (33%) and Mauritania (15%). The authors should consider to mention this and the possibility to add this type of information in the future releases because it can be very relevant for countries with a minimal electrical infrastructure.

***Author Response: **We agree with the reviewer that this is important information to include in the database. We have released a new version of the database which includes this information as metadata (columns « SharedPlant » and « SharedPlantSplit » on worksheet no. 1 of the database).*  

- Do the authors have tried to validate the seasonal patterns of the capacity factors? For example, some Transmission System Operators in African countries publish information on their hydropower plants.

***Author Response: **In general, there is ample data available to validate annual average capacity factors (see the « Availability » column in the AHA results sheets), but seasonal patterns are hard to come by from publicly available information. Therefore, while such an exercise could be performed for some individual plants for which such information is publicly available (for a limited set of years), the authors of this paper currently lack temporal and budgetary resources to obtain such data from all African TSOs to provide a comprehensive validation. However, the authors are interested in performing such an exercise, and hope that this paper may stimulate data sharing to facilitate this.*  

- The data published is very rich but the text files lack metadata. The authors should add: a) information on the meaning of the columns in the txt files and b) information on the linkage between the Excel file and the SWAT data, in other words what the link between items in SWAT_reservoir_mon_EWEMBI_hist.txt and the plants in the Excel file? I think the third point is very important to make the data published more usable and accessible. I would also invite the authors of using a more user-friendly format for their data in the future, for example considering creating a Tabular Data Package.

***Author Response: **We thank the reviewer for this comment. We take the opportunity to clarify that the link between the items in the .txt files and the plants in the Excel file is clarified by columns “River Channel” and “Reservoir ID”. Each hydropower plant location is linked to a unique River Channel ID and (if existing reservoir present) Reservoir ID. The AHA code, deposited on GitHub, reads the River/Reservoir ID from the Excel file and extracts the corresponding river flow dataset from SWAT+ in the .txt file. It thus links each hydropower plant to its corresponding river flow.* *Concerning the data format, we note that the .txt files are provided as input to run the simulations on and given in a format that allows to seamlessly integrate SWAT+ output with AHA processing (the SWAT+ model provides output in .txt format which is subsequently read in by the AHA code). The data product of this paper is the Excel file that represents the African Hydropower Atlas. We note that the .txt files contain too many rows to be presented in tabular (.csv) format.* *We refer to the SWAT+ output documentation (accessible through* *https://swatplus.gitbook.io/docs/download-docs* *) for further metadata on the columns included in this .txt file.*
